# Molecular characterisation defines clinically-actionable heterogeneity within Group 4 medulloblastoma and improves disease risk-stratification

**DOI:** 10.1007/s00401-023-02566-0

**Published:** 2023-04-04

**Authors:** Jack Goddard, Jemma Castle, Emily Southworth, Anya Fletcher, Stephen Crosier, Idoia Martin-Guerrero, Miguel García-Ariza, Aurora Navajas, Julien Masliah-Planchon, Franck Bourdeaut, Christelle Dufour, Olivier Ayrault, Tobias Goschzik, Torsten Pietsch, Martin Sill, Stefan M. Pfister, Stefan Rutkowski, Stacey Richardson, Rebecca M. Hill, Daniel Williamson, Simon Bailey, Edward C. Schwalbe, Steven C. Clifford, Debbie Hicks

**Affiliations:** 1 Wolfson Childhood Cancer Research Centre, Newcastle University Centre for Cancer, Translational and Clinical Research Institute, Newcastle University, Newcastle Upon Tyne, UK; 2 Biocruces Bizkaia Health Research Institute, Barakaldo, Spain; 3 Department of Genetics, Physical Anthropology and Animal Physiology, University of the Basque Country, Leioa, Spain; 4 Department of Pediatric Hematology and Oncology, Cruces University Hospital, Barakaldo, Spain; 5 Unité de Génétique Somatique, Institut Curie, Paris, France; 6 SIREDO Pediatric Oncology Center, Curie Institute, Paris, France; 7 Department of Pediatric and Adolescent Oncology, Gustave Roussy, Rue Edouard Vaillant, 94805 Villejuif, France; 8 UMR 3347, INSERM U1021, Institut Curie, PSL Research University, Université Paris Sud, Université Paris-Saclay, CNRS, Paris, France; 9 Department of Neuropathology, DGNN Brain Tumour Reference Center, University of Bonn Medical Center, Bonn, Germany; 10 Hopp Children’s Cancer Center Heidelberg (KiTZ), Heidelberg, Germany; 11 Division of Paediatric Neurooncology, German Cancer Research Center (DKFZ) and German Cancer Consortium (DKTK), Heidelberg, Germany; 12 Department of Paediatric Haematology and Oncology, Heidelberg University Hospital, Heidelberg, Germany; 13 Department of Pediatric Hematology and Oncology, University Medical Center Hamburg-Eppendorf, Hamburg, Germany; 14 Department of Applied Sciences, Northumbria University, Newcastle Upon Tyne, UK

**Keywords:** Paediatric Oncology, Medulloblastoma, Risk-stratification, Biomarkers

## Abstract

Group 4 tumours (MB_Grp4_) represent the majority of non-WNT/non-SHH medulloblastomas. Their clinical course is poorly predicted by current risk-factors. MB_Grp4_ molecular substructures have been identified (e.g. subgroups/cytogenetics/mutations), however their inter-relationships and potential to improve clinical sub-classification and risk-stratification remain undefined. We comprehensively characterised the paediatric MB_Grp4_ molecular landscape and determined its utility to improve clinical management. A clinically-annotated discovery cohort (n = 362 MB_Grp4_) was assembled from UK-CCLG institutions and SIOP-UKCCSG-PNET3, HIT-SIOP-PNET4 and PNET HR + 5 clinical trials. Molecular profiling was undertaken, integrating driver mutations, second-generation non-WNT/non-SHH subgroups (1–8) and whole-chromosome aberrations (WCAs). Survival models were derived for patients ≥ 3 years of age who received contemporary multi-modal therapies (n = 323). We first independently derived and validated a favourable-risk WCA group (WCA-FR) characterised by ≥ 2 features from chromosome 7 gain, 8 loss, and 11 loss. Remaining patients were high-risk (WCA-HR). Subgroups 6 and 7 were enriched for WCA-FR (p < 0·0001) and aneuploidy. Subgroup 8 was defined by predominantly balanced genomes with isolated isochromosome 17q (p < 0·0001). While no mutations were associated with outcome and overall mutational burden was low, WCA-HR harboured recurrent chromatin remodelling mutations (p = 0·007). Integration of methylation and WCA groups improved risk-stratification models and outperformed established prognostication schemes. Our MB_Grp4_ risk-stratification scheme defines: favourable-risk (non-metastatic disease and (i) subgroup 7 or (ii) WCA-FR (21% of patients, 5-year PFS 97%)), very-high-risk (metastatic disease with WCA-HR (36%, 5-year PFS 49%)) and high-risk (remaining patients; 43%, 5-year PFS 67%). These findings validated in an independent MB_Grp4_ cohort (n = 668). Importantly, our findings demonstrate that previously established disease-wide risk-features (i.e. LCA histology and *MYC(N)* amplification) have little prognostic relevance in MB_Grp4_ disease. Novel validated survival models, integrating clinical features, methylation and WCA groups, improve outcome prediction and re-define risk-status for ~ 80% of MB_Grp4_. Our MB_Grp4_ favourable-risk group has MB_WNT_-like excellent outcomes, thereby doubling the proportion of medulloblastoma patients who could benefit from therapy de-escalation approaches, aimed at reducing treatment induced late-effects while sustaining survival outcomes. Novel approaches are urgently required for the very-high-risk patients.

**Supplementary Information:**

The online version contains supplementary material available at 10.1007/s00401-023-02566-0.

## Introduction

Medulloblastoma (MB) is the most common malignant embryonal tumour of the central nervous system (CNS, WHO grade 4) in children accounting for approximately 10% of all paediatric cancer deaths. Current multi-modal treatments for non-infants comprise surgical resection and cranio-spinal radiation (CSI), followed by adjuvant chemotherapy [26]. These treatments commonly cause long-term neurological, neurocognitive and neuroendocrine deficits as well as increased risk for second malignancies [6].

MB comprises molecular disease groups which form the basis of the genetically-defined medulloblastoma classification in the 2021 WHO classifications of CNS tumours, alongside its histologically-defined MB entities. These are: WNT-activated (MB_WNT_), Sonic Hedgehog-activated (MB_SHH_) *TP53*-wildtype, MB_SHH_ *TP53*-mutant and non-WNT/non-SHH (comprising Group 3 (MB_Grp3_) and Group 4 (MB_Grp4_)) [19].

Current clinical risk-stratification models for MB use established clinical and pathological risk-features derived from studies of disease-wide cohorts. Metastatic disease, sub-total surgical resection, and large cell/anaplastic (LCA) histology have long been associated with poor outcomes in such studies [16, 36]. Alongside these, molecular features have profoundly improved our ability to predict risk in MB. MB_WNT_ patients aged 3–16 years old consistently achieve favourable outcomes (> 95% 5-year progression free survival) [7, 12, 28] and, within MB_SHH_, *TP53* mutations are associated with an extremely poor prognosis [38]. In addition, amplification of either the *MYC* or *MYCN* oncogenes have strong associations with inferior survival outcomes and are associated with other high-risk features [31]. Together, these risk-features underpin treatment stratifications in current international biomarker-driven clinical trials, which reduce therapy for favourable MB_WNT_ and use intensified regimens for patients with high-risk features (SIOP-PNET5-MB [21] [NCT02066220], SIOP-HR-MB [2] [EudraCT Number: 2018-004250-17], SJMB012 [NCT01878617]). However, these disease-wide risk-features show molecular group dependency (e.g. *MYC* is prognostic in MB_Grp3_; *MYCN* in MB_SHH_) and current disease-wide risk-stratification models do not adequately characterise risk specifically within MB_Grp4_, which accounts for ~ 40% of MB patients and the majority of non-WNT/non-SHH cases. The identification and validation of prognostic biomarkers, which could direct risk-adapted adjuvant therapies for MB_Grp4_ patients, thus represents an urgent unmet clinical need.

Molecular substructure within MB_Grp3_ and MB_Grp4_ was recently described in three independent studies, each proffering different solutions [3, 23, 33, 34]. Cavalli et al. identified that MB_Grp3_ and MB_Grp4_ each partition into three subgroups (α, β, and γ) with specific transcriptional profiles [3], whilst Northcott et al. identified and mutationally characterised eight subgroups (I-VIII) that are shared by MB_Grp3/Grp4_ [23]. Schwalbe et al. highlighted the prognostic relevance of MB_Grp3_ and MB_Grp4_ molecular substructure by defining clinically relevant high- and low-disease risk subgroups (MB_Grp3-LR/HR,_ MB_Grp4-LR/HR_) [33]. A subsequent international meta-analysis resolved this disparity and supported a definition comprising eight robust, clinically-relevant, second-generation methylation MB_Grp3/Grp4_ subgroups (1–8) [34]; these second-generation methylation subgroups have been adopted into the 2021 WHO classification of CNS tumours [19] and their clinical behaviour has been further confirmed in contemporary, clinically controlled, cohorts [14, 22]. Furthermore, transcriptomic profiling of MB_Grp3/Grp4_ has also shown that they can be represented as a bipolar continuum between archetypical MB_Grp3_ and MB_Grp4_, denoted by a MB_Grp3/Grp4_ continuum score [37]. Second-generation methylation subgroups can be ordered on this expression continuum from prototypic MB_Grp4_ subgroups (8, 7 and 6), through mixed subgroups (5, 1), to MB_Grp3_ subgroups (3, 4, 2).

The underlying biology of MB_Grp4_ is complex. In contrast to MB_WNT_ and MB_SHH_, MB_Grp4_ has a relative paucity of defining genetic drivers; analysis of gene-specific mutations and their clinical relevance has not yet been undertaken in large clinically-annotated MB_Grp4_ cohorts. Cytogenetically-defined prognostic features have been described within MB_Grp4_, first as specific whole-chromosome aberrations (11 loss or 17 gain) [35] and, more recently, through the recognition of a concerted whole chromosomal aberration (WCA) signature within the clinically defined standard-risk (i.e. non-metastatic, non-LCA and non-*MYC* amplified) HIT-SIOP-PNET4 trial [NCT01351870] non-WNT/non-SHH MB cohort. This signature (defined by ≥ 2 of whole-chromosome 7 gain, chromosome 8 loss, and chromosome 11 loss (WCA-FR)) is associated with increased ploidy, multiple non-random WCAs, and predicted a favourable prognosis in both trial and validation cohorts (5-year PFS, 100%). Remaining tumours (WCA-HR) had much poorer outcomes (68% 5-year PFS) [16]. Mynarek et al. recently incorporated WCA signatures as risk stratification parameters in data from the German Paediatric Brain Tumour (HIT) trials [22].

Importantly, inter-relationships between the different MB_Grp4_ molecular characteristics (i.e. second-generation subgroups, cytogenetic groups, mutations), their utility and performance as prognostic biomarkers, and potential for integration into risk-stratification schemes, remains to be established. Previous studies of MB_Grp4_ have been limited by cohort size and/or comprehensive clinical annotation [3, 23, 24, 34, 35] and have not permitted the integrated characterisation of MB_Grp4_ molecular pathology, alongside assessment of its translational potential to improve clinical sub-classification and risk-stratification.

Here, we report a comprehensive characterisation of the molecular pathology of primary MB_Grp4_, integrating disease subgroups, mutational and copy-number events, and assess their translational potential in large, clinically-annotated discovery and validation cohorts, comprising > 1000 MB_Grp4_ tumours. We describe non-random, clinically-actionable biological heterogeneity, which forms the basis of novel biomarker-driven risk-stratification models. These models improve outcome prediction and reassign risk-status for ~ 80% of MB_Grp4_ patients. These findings refine our understanding of MB_Grp4_ biology and provide a foundation for personalised therapies, improved therapeutic strategies and future clinical trials.

## Materials and methods

### Study design and participants

A discovery cohort of 362 primary MB_Grp4_ tumours (Supplementary Fig. S1a, online resource) and a comparator cohort of 489 non-MB_Grp4_ tumours (Supplementary Table 1, online resource) were assembled from UK-Children’s Cancer and Leukaemia Group (CCLG) institutions, collaborating international centres and the SIOP-UKCCSG-PNET3 [11], HIT-SIOP-PNET4 [16], and PNET HR + 5 [10] clinical trials (Supplementary Fig. S1a, online resource). Furthermore, 668 independent MB_Grp4_ samples from two published studies [3, 23] were used as validation cohorts for clinical and molecular features (Supplementary Fig. S1a, online resource).

Ethical approval and consents were given.

### Procedures

For the discovery and non-MB_Grp4_ comparator cohorts, MB diagnosis was confirmed by methylation-based classification [33] and/or central neuropathology review (CPR, 81%) which provided histological sub-classification. In the absence of CPR, institutional annotation was used. Metastatic staging was assigned according to Chang’s criteria (M + ; M stages 1–4, M0; local disease only) [4]. Extent of resection was evaluated institutionally and tumours were considered sub-totally resected if residual disease exceeded 1·5cm^2^ [1].

Molecular groups were assigned using established methods, and only tumours confidently assigned as MB_Grp4_ were included in the discovery cohort [32, 33], Second-generation methylation subgroups were assigned using the ‘MNP Medulloblastoma classifier group 3/4’ version 1·0 at www.molecularneuropathology.org/mnp. Chromosome arm-level copy number estimates were derived from Illumina HumanMethylation 450K/EPIC array data, using the package ‘Conumee’ (R/Bioconductor) as previous described [30, 33]. Molecular inversion probe (MIP) array was used for the HIT-SIOP-PNET4 cohort to call arm-level copy number as previously described [16]. *MYC*(*N*) copy number status was assessed by iFISH, copy number estimates from methylation array and/or MLPA [17]. Established non-WNT/non-SHH focal copy number variants (CNVs, Supplementary Table 2, online resource) were assessed as described [27, 30].

Targeted gene panel (n = 168) and whole-exome (n = 4) sequencing was carried out to interrogate the mutational status of 63 putative MB driver genes (Supplementary Table 2, online resource). *KBTBD4* mutations in the Kelch motif were assessed by Sanger sequencing [5, 23]. *PRDM6* and *GFI1/1B* expression was evaluated by RNA-sequencing using established methods [23, 25, 33].

### Statistical and survival analysis

In accordance with current treatment protocols, survival analysis in the discovery cohort was restricted to patients aged ≥ 3 years who received CSI and where outcome data was available (323/362 [89%]). Univariable and multivariable Cox proportional hazards models were used to investigate predictors of progression free survival (PFS). Multivariable Cox models (n = 213 patients with available subgroup data) were constructed using backwards selection, considering established clinico-molecular and treatment variables (metastatic disease, extent of resection, LCA pathology, sex, *MYC*/*MYCN* amplification, i17q, and dose of CSI) in addition to biologically and clinically significant molecular factors (WCA status, subgroup 7, chromosome 13 loss, subgroup 5, chromosome 18 gain). To assess performance, bootstrapped models were generated using 1000 rounds of resampling to assess calibration and discrimination at 5 years from diagnosis and were tested in an independent validation cohort [23]. From the multivariable Cox model, a novel, clinically-deliverable MB_Grp4_ risk-stratification scheme was generated from combinations of markers by categorising patients using selected variables into risk groups with established disease cut-offs for projected 5-year PFS (favourable-risk, > 90%; standard-risk, > 75–90%; high-risk, 50–75%; very-high-risk, < 50%) [29]. We finally compared our risk-stratification scheme to those in current clinical practice [2, 21] and previously reported molecular stratification schemes [15, 35] by assessing discrimination and calibration performance in the discovery and independent validation cohorts, once again using 1000 rounds of resampling and measuring at 5 years from diagnosis. Proportionality of covariates in Cox modelling were tested using scaled Schoenfeld residuals. Kaplan–Meier curves with log-rank tests were constructed to visualise survival associations.

Fisher’s exact and Chi squared tests were used to assess associations between categorical variables. Kruskal–Wallis, Mann–Whitney U, ANOVA and t-tests were used to compare continuous variables between groups. Significant associations were defined as having an adjusted p value of < 0·05 using the Benjamini–Hochberg procedure to correct for multiple testing. Statistical and bioinformatics analyses were done using R statistical environment (version 4.0.4).

Full methodological detail can be found within the Supplementary Material (online resource).

### Role of the funding source

The funders had no role in study design.

## Results

As anticipated, MB_Grp4_ tumours typically arose in older children (median 8 years [0·2–20], p = 0·0002), displayed classic pathology (n = 277/328 [84%], p < 0·0001), and were enriched for isochromosome 17q (i17q) (n = 196/353 [56%], p < 0·0001; Supplementary Table 1, online resource) compared to a non-MB_Grp4_ comparator cohort [26]. Subgroup 8 predominated (n = 92/248 [37%]), followed by subgroup 7 (n = 73/248 [29%]), subgroup 6 (n = 38/248 [15%]), subgroup 5 (n = 36/248 [14%]) and subgroup 1 (n = 9/248 [3%]; Table 1, Supplementary Fig. S4a, online resource). Outside of WCAs, *PRDM6* overexpression was the most common molecular event (n = 18/83 [22%]; Supplementary Fig. S1c, online resource). We found low frequencies of mutations and focal CNVs, however these converged on common biological ontologies (Supplementary Table 2, online resource). Aberrations in genes involved in transcriptional regulation (30%) were most frequent followed by mutations in chromatin remodelling (29%) genes; incorporating *KMT2C* (n = 16/172 [9%]), *KMT2D* (n = 13/172 [8%]), *KDM6A* (n = 13/182 [7%]) and *ZMYM3* (n = 12/172 [7%])) with coalescing functions as modifiers of H3K4 and H3K27-methylation. Aberrations in genes involved in genome maintenance (13%) and PI3K/AKT signalling (6%) were also observed (Supplementary Fig. S1c, online resource). No detectable mutation was seen in ~ 20% of patients.

**Table 1:** Clinicopathological and molecular features of our discovery (n = 362) and validation (n = 668) cohorts

	Discovery cohort (n = 362)	Validation cohort (n = 668)
Age at diagnosis (years)		
Median (range)	8.0 (0.2–20)	8·4 (1–20)
Sex		
Male	242 (67%)	327 (72%)
Female	119 (33%)	129 (28%)
M:F ratio	2.0:1	2.5:1
No Data	1	212
Resection		
GTR	242 (72%)	NA
STR	92 (28%)	
No Data	28	
Histology		
CLA	277 (84%)	240 (86%)
DN/MBEN	30 (9%)	26 (9%)
LCA	21 (6%)	14 (5%)
MBNOS	17	–
No Data	17	388
*MYC* amplification		
Yes	6 (2%)	7 (1%)
No	327 (98%)	611 (99%)
No Data	29	–
*MYCN* amplification		
Yes	24 (7%)	30 (4%)
No	308 (93%)	638 (96%)
No data	30	–
Metastatic disease		
M +	111 (34%)	185 (50%)
M0	212 (66%)	186 (50%)
No data	39	297
i17q		
Yes	196 (56%)	364 (55%)
No	157 (44%)	304 (45%)
No data	9	–
WCA group		
WCA-FR	105 (30%)	156 (23%)
WCA-HR	248 (70%)	512 (77%)
No Data	9	–
Second-generation methylation subgroups		
1	9 (4%)	18 (3%)
5	36 (15%)	51 (8%)
6	38 (15%)	85 (14%)
7	73 (29%)	196 (33%)
8	92 (37%)	252 (42%)
Not classified	31	66
No data	83	–
Radiotherapy		
CSI	324 (96%)	NA
Focal	9 (3%)	
Not given	6 (2%)	
No data	23	
CSI dose		
HD RTX	205 (66%)	NA
SD RTX	104 (34%)	
No data	23	
Chemotherapy		
Yes	316 (93%)	NA
No	23 (7%)	
No data	23	
Chemotherapy dose		
HD CTX	47 (16%)	NA
SD CTX	255 (84%	
No data	90	
Median follow-up time (years)		
PFS (IQR)	6.64 (5·50–8·50)	6.21 (4.50–9.16)^*^
OS (IQR)	6.55 (5·12–8·85)	4·83 (2.70–9.00)^#^
5-year PFS and OS		
PFS (95% CI)	68% (0·63–0·73)	71% (0.65–0.79)^*^
OS (95% CI)	73% (0·68–0·79)	78% (0.71–0.85)^#^

Data are n (%) or median (range). *GTR* gross total resection, *STR* = sub-total resection, *CLA* = classic, *DN/MBEN* = desmoplastic/nodular or medulloblastoma with extensive nodularity, *LCA* large-cell/anaplastic, MBNOS medulloblastoma not otherwise specified, *M +*  metastatic disease, *M0*  non-metastatic disease, *i17q* isochromosome 17q, *WCA-FR/HR*  whole chromosome aberration-favourable risk/high risk, *CSI*  craniospinal irradiation, *RTX* = radiotherapy, *CTX*  chemotherapy, *HD*  high dose, *SD*  standard dose, *PFS*  progression-free survival, *OS*  overall survival, *NA* not applicable. For the validation survival cohorts, these were considered separately and consisted of either samples from Northcott et al. [23] ^*^ or Cavalli et al. [3] ^#^ due to availability of survival data for only PFS or OS in each cohort, respectively

In contrast, multiple recurrent arm-level and WCAs were common (Supplementary Fig. S1d, online resource). We sought to re-derive, from first principles, prognostic WCA signatures [16] in our risk-independent all-comer MB_Grp4_ cohort (Supplementary Fig. S2, online resource). Analysis recapitulated the previous finding that two or more of chromosome 7 gain, chromosome 8 loss and chromosome 11 loss (WCA-FR) represented the optimum combination of WCAs for predicting PFS (Supplementary Fig. S2i, online resource). Unsupervised hierarchical clustering and association analysis further supported two distinct WCA signatures within MB_Grp4_; i17q negatively associated with other WCAs, while chromosome 7 gain, 8 loss and 11 loss positively associated with most other WCAs (Supplementary Fig. S3a, b, online resource).

**Fig. 1:**
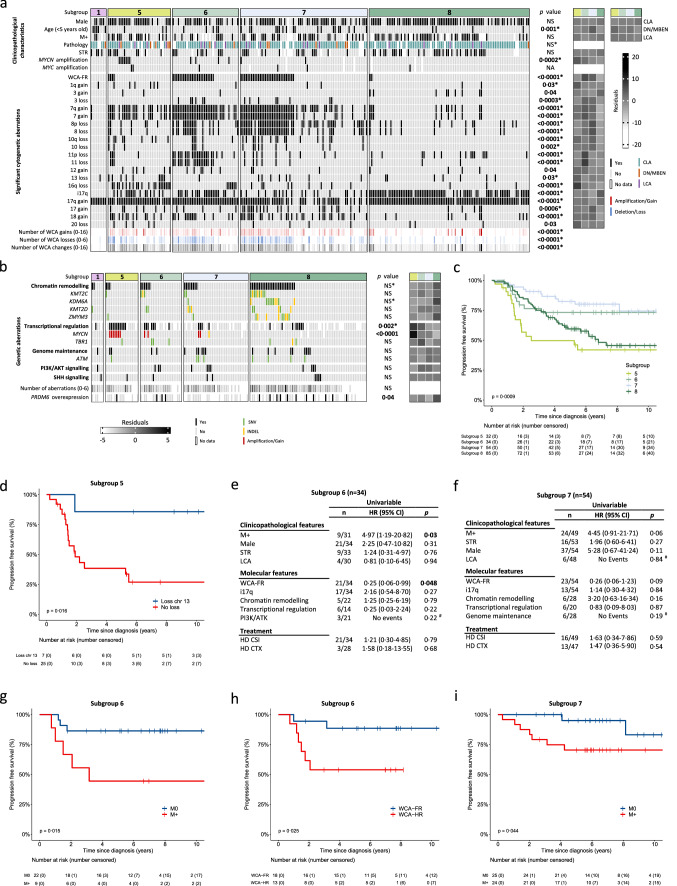
Characterisation of MB_Grp4_ second-generation methylation subgroups. Distribution of **a** established clinicopathological characteristics and significantly enriched cytogenetic aberrations. **b** Gene-specific genetic alterations within MB_Gpr4_ methylation subgroups. For Fig. 2a and b, significance is shown from Fisher’s exact or Kruskal–Wallis tests, *depicts significance recapitulated in validation cohort. Residuals from *χ*^2^ test indicate subgroup-enrichment (strong relationships are indicated by darker shades of grey) alongside scale bar. Number of WCA gains (red), losses (blue), total WCAs and number of genetic aberrations (black) are also shown with increasing colour intensity indicating a higher number of changes. Features with a cohort-wide frequency of ≥ 5% or with a subgroup-specific frequency ≥ 10% were included in the analysis. *MYC* amplifications are shown despite their low frequency. Full data is shown in Supplementary Fig. S4, online resource. **c** Kaplan–Meier plot of PFS by MB_Grp4_ methylation subgroup. **d** Kaplan–Meier plot for PFS in subgroup 5 for chromosome 13 loss. Univariable Cox proportional hazards models of PFS in MB_Grp4_ **e** subgroup 6 and **f** subgroup 7 for clinical and molecular features ≥ 10%. Kaplan–Meier plots of PFS by **g** metastatic disease in subgroup 6, **h** WCA groups in subgroup 6 and **i** metastatic disease in subgroup 7. At-risk tables are shown in two-year increments with number of patients censored in parentheses with significance shown by p value generated from log-rank test. Abbreviations: *M + * metastatic disease, *M0* non-metastatic disease, *STR*  sub-total resection, *CLA* classic, *DN/MBEN* desmoplastic/nodular or medulloblastoma with extensive nodularity, *LCA* large-cell/anaplastic, *WCA-FR/HR* whole chromosome aberrations-favourable risk/high risk, *CTX* chemotherapy, *HR* hazard ratio, *CI* confidence interval. ^#^Estimates not possible due to group with no events, p values reported from log-rank test

We next considered molecular and clinical heterogeneity within MB_Grp4_, first focusing on methylation subgroups. While patients < 5 years are uncommon in MB_Grp4_ overall, subgroup 7 harboured a significant enrichment in these youngest patients (n = 19/72 [26%], p = 0·001; Fig. 1a, Supplementary Fig. S4b, c, online resource). There was no significant association with extent of surgical resection or metastatic disease (Fig. 1a, Supplementary Fig. S4c, online resource).

MB_Grp4_ methylation subgroups displayed distinct cytogenetic profiles (Fig. 1a, Supplementary Fig. S4c, online resource). Subgroup 5 harbours distinct changes including both 16q loss (21/36, 58%; p < 0·0001) and 13 loss (9/36, 25%; p = 0·03). Subgroups 6 and 7 were highly disrupted cytogenetically, with multiple WCAs (gains/losses; median = 3·5 [IQR 2–5] and 4 [IQR 1·5–6], respectively) and were enriched for the WCA-FR group (subgroup 6, n = 24/38 [63%] and subgroup 7 n = 31/73 [42%], p < 0·0001). Whilst chromosome 7 gain and chromosome 8 loss were equally distributed between subgroups 6 and 7, chromosome 11 loss was largely restricted to subgroup 6 (subgroup 6 n = 19/38 [50%] and subgroup 7 n = 6/73 [8%], p < 0·0001). In contrast, subgroup 8 showed a quiet cytogenetic profile with a lower total number of WCAs (median = 1 [IQR 1–2]) and i17q commonly its sole defining feature (n = 71/92 [77%], p < 0·0001).

Mutations and focal CNVs were less characteristic of subgroups than WCAs (Fig. 1b, Supplementary Fig. S4d, online resource). Subgroup 8 harboured mutations in chromatin remodelling genes: *KDM6A* (10/11 mutations detected occurred in subgroup 8, 91%), *ZMYM3* (9/11, 81%), *KMT2C* (9/15, 60%) and *KMT2D* (5/10, 50%) occurred at higher frequencies, although these were not significant. Alterations in genes with overlapping functions relating to transcriptional regulation showed a significant association with subgroup 5 (p = 0·002), primarily driven by the high frequency of *MYCN* aberrations (p < 0·0001). Lastly, high *PRDM6* expression showed significant enrichment within subgroup 8 (p = 0·04; Fig. 1b). The described enrichments of clinico-molecular features validated (Supplementary Fig. S4c, d, online resource) in an independent cohort.

MB_Grp4_ methylation subgroups also showed distinct survival outcomes (Fig. 1c). Subgroups 6 and 7 were associated with superior survival outcomes (subgroup 6; 5-year PFS 73%, 60–90 [95% CI], subgroup 7; 5-year PFS 82%, 73–94 [95% CI]). In comparison, subgroups 5 and 8 had poorer survival outcomes (subgroup 5; 5-year PFS 50%, 34–71 [95% CI], subgroup 8; 5-year PFS 59%, 50–71 [95% CI]).

**Fig. 2:**
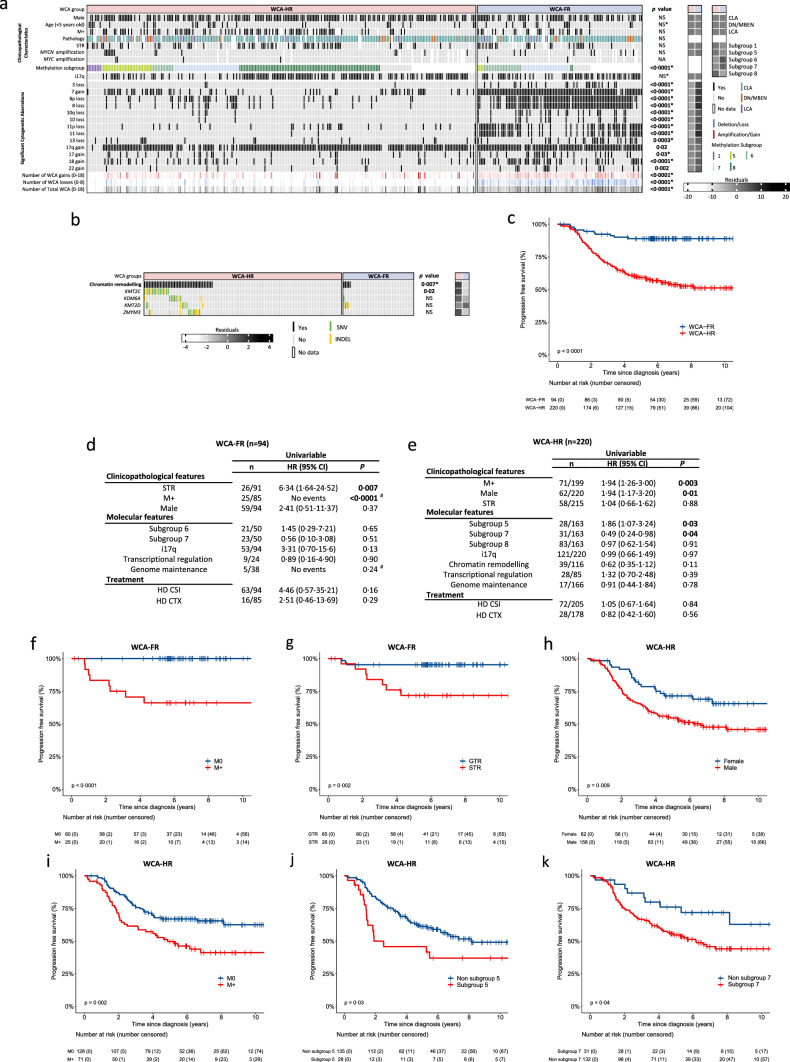
Characterisation of MB_Grp4_ WCA groups. Distribution of **a** established clinicopathological characteristics and significant cytogenetic aberrations. **b** Genetic alterations in chromatin remodelling genes within MB_Gpr4_ WCA groups. For **a** and **b**, significance is shown from Fisher’s exact or Mann–Whitney U tests, *depicts significance recapitulated in validation cohort. Residuals from χ^2^ test indicate subgroup-enrichment (strong relationships are indicated by darker shades of grey) alongside scale bar. Number of WCA gains (red), losses (blue), and total WCA (black) are also shown, with increasing colour intensity indicating a higher number of changes. Features with a cohort-wide frequency of ≥ 5% or with a subgroup-specific frequency ≥ 10% were included in the analysis. *MYC* amplifications are shown despite low frequency. All data is shown in Supplementary Fig. S5, online resource. **c** Kaplan–Meier plot of PFS by MB_Grp4_ WCA groups. Univariable Cox proportional hazards models of PFS within **d** WCA-FR and **e** WCA-HR, assessing clinical and molecular features ≥ 10%. Kaplan–Meier plot of PFS by **f** metastatic disease and **g** extent of resection within WCA-FR. Kaplan–Meier plot of PFS by **h** sex, **i** metastatic disease, **j** subgroup 5 and **k** subgroup 7 within WCA-HR. At risk tables are shown in two-year increments with number of patients censored in parentheses with significance shown by p value generated from log-rank test. Abbreviations: *M + * metastatic disease, *M0* non-metastatic disease, *STR*  sub-total resection, *CLA* classic, *DN/MBEN* desmoplastic/nodular or medulloblastoma with extensive nodularity, *LCA* large-cell/anaplastic, *i17q* isochromosome 17q, *WCA-FR/HR* whole chromosome aberrations-favourable risk/high risk, *CTX* chemotherapy, *HR* hazard ratio, *CI* confidence interval. ^#^Estimates not possible due to group with no events, p values reported from log-rank test

Survival analysis was carried out to further identify risk-features within MB_Grp4_ methylation subgroups. Chromosome 13 loss was the sole significant predictor of improved PFS within subgroup 5 (HR 0·12, 0·02–0·94 [95% CI], p = 0·04; Supplementary Fig. S4e, online resource; log-rank test, p = 0·016, Fig. 1d). Univariable Cox regression within subgroup 6 (Fig. 1e, 2g, 2h) identified that metastatic disease was associated with poor PFS (HR 4·97, 1·19–20·82 [95% CI], p = 0·03). WCA-FR was associated with improved PFS within subgroup 6 at large (HR 0·25, 0·06–0·99 [95% CI], p = 0·048) or within a M0 restricted cohort (Supplementary Fig. S5a, online resource). Within subgroup 7, metastatic status was marginally significant (HR 4·45, 0·91–21·71 [95% CI], p = 0·06; Fig. 1f, p = 0·04, log-rank test; Fig. 1i). WCA-FR did not associate with improved PFS in subgroup 7, irrespective of metastatic disease status (Supplementary Fig. S5b, c, online resource). Survival heterogeneity in subgroup 8 could not be explained by any clinico-molecular features tested (Supplementary Fig. S4f, online resource).

We next defined the cytogenetic/mutational landscape of the validated MB_Grp4_ WCA groups originally identified in the standard-risk HIT-SIOP-PNET4 cohort [16], and explored their clinical behaviour in our risk-independent all-comers’ cohort. WCA-FR strongly associated with subgroup 6 and 7 (p < 0·0001) and was cytogenetically complex, harbouring many significantly enriched WCAs: whole-chromosome 3 loss, 7 gain, 8 loss, 10 loss, 11 loss, 13 loss, 17 gain, 18 gain and 22 gain. The number of WCA gains/losses and total WCAs differed significantly (p < 0·0001), with WCA-HR having few changes (median = 2 [IQR 1–3]) compared to WCA-FR (median = 6 [IQR 4–8]) (Fig. 2a, Supplementary Fig. S6a, online resource).

The WCA-HR group was cytogenetically quiet, with i17q in isolation commonly representing the single defining cytogenetic feature (Fig. 2a, Supplementary Fig. S6a, online resource). Although the total mutational burden was equivalently low for both groups (Supplementary Fig. S6b, online resource), the WCA-HR group was significantly enriched for mutations in chromatin remodelling genes (p = 0·007); mutations in *KMT2C* (p = 0·02) and *ZMYM3* (not significant) were exclusive to WCA-HR and mutations in other chromatin remodelling genes (*KDM6A* (10/11 mutations occurred in the WCA-HR group, 91%) and *KMT2D* (8/12 mutations, 67%)), were also present at high frequencies (Fig. 2b). The majority of these features validated (Supplementary Fig. S6a, b, online resource) in an independent cohort. Established clinico-molecular risk-features (age at diagnosis, metastatic disease, histological variants, extent of surgical resection and amplification of *MYCN*) were equivalently distributed between both groups (Fig. 2a, Supplementary Fig. S6a, online resource).

The WCA-FR group (n = 94/314 [30%]) conferred excellent outcomes with a 5-year PFS of 89% (0·83–0·96 [95% CI]) compared with the WCA-HR group (n = 220/314 [70%]), which performed worse with a 5-year PFS of 59%, (0·53–0·66 [95% CI], p < 0·0001; Fig. 2c). This survival relationship validated in an external cohort: WCA-FR (n = 46/191 [24%]) 5-year PFS of 98% (0·94–1·00 [95% CI]) compared with the WCA-HR group (n = 145/191 [76%]) 5-year PFS of 73% (0·65–0·81 [95% CI]; Supplementary Fig. S6c, online resource).

Univariable survival analysis was carried out within WCA-FR and WCA-HR groups. Metastatic disease (log-rank p < 0·0001; log rank was used as WCA-FR M0 patients had no events) and STR (HR 6·34, 1·64–24·52 [95% CI], p = 0·007) was significantly associated with poorer PFS within WCA-FR (Fig. 2d, f, g); non-metastatic patients showed extremely favourable survival outcomes (5-year PFS 100%). Within WCA-HR, metastatic disease (HR 1·94, 1·23–3·00 [95% CI], p = 0·003), male sex (HR 1·94, 1·17–3·20 [95% CI], p = 0·01) as well as subgroup 5 (HR 1·86, 1·07–3·24 [95% CI], p = 0·03) were prognostic for poor outcome (Fig. 2e, h, i, j). Subgroup 7 (HR 0·49, 0·24–0·98 [95% CI], p = 0·04) was associated with improved outcomes (Fig. 2e, k).

**Fig. 3:**
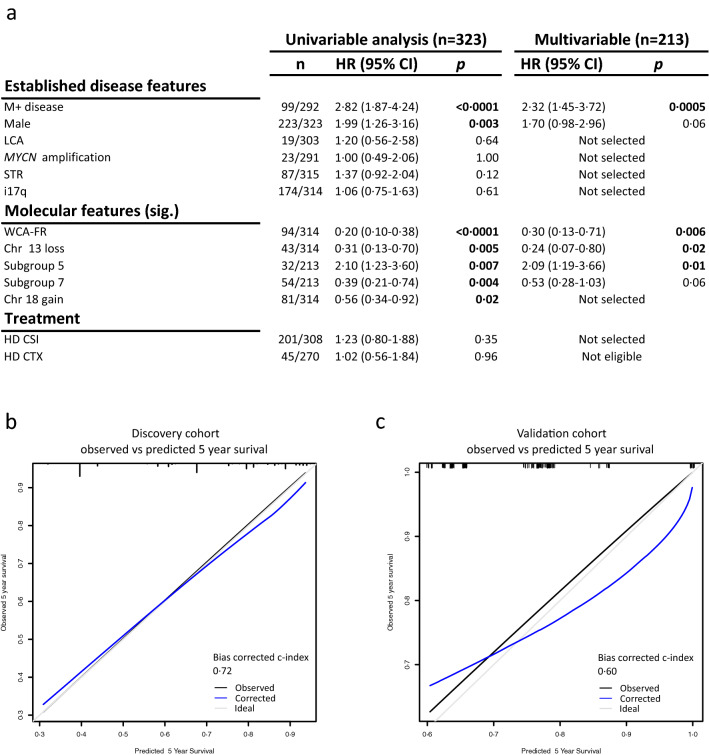
Identification of prognostic survival markers within MB_Grp4_. **a** Univariable (n = 323) and multivariable (n = 213) Cox regression analyses of progression-free survival in our MB_Grp4_ survival cohort. Established MB features were considered alongside molecular factors with a frequency ≥ 10%; only significant features (sig.) are shown. For multivariable analysis, HR and p values are shown for variables retained from backwards selection. Dose of chemotherapy was not considered due to extent of missing data. Calibration plots of multivariable Cox models within both **b** discovery and **c** validation cohorts for survival at 5 years alongside the bias-corrected c-index. Abbreviations: *M + * metastatic disease, *M0* non-metastatic disease, *STR*  sub-total resection, *CLA* classic, *DN/MBEN* desmoplastic/nodular or medulloblastoma with extensive nodularity, *LCA* large-cell/anaplastic, *WCA-FR/HR* whole chromosome aberrations-favourable risk/high risk, *CTX* chemotherapy, *HR* hazard ratio, *CI* confidence interval

To assess predictors of risk across MB_Grp4_, we assessed all eligible [see methods] clinical, pathological and molecular features (i.e. subgroups, focal and arm-level CNVs, mutations), in univariable Cox regression analysis and found multiple significant associations (Fig. 3a). For multivariable Cox regression analysis, we selected established MB features (metastatic disease, LCA histology, STR, sex, i17q, *MYCN* amplification and CSI dose) as well as biologically and clinically significant molecular factors (WCA status, subgroup 5, subgroup 7, chromosome 13 loss, chromosome 18 gain). In the multivariable analysis, metastatic disease (HR 2·32, 1·45–3·72 [95% CI], p = 0·0005), WCA-FR (HR 0·30, 0·13–0·71 [95% CI], p = 0·006), subgroup 5 (HR 2·09, 1·19–3·66 [95% CI], p = 0·01), subgroup 7 (HR 0·53, 0·28–1·03 [95% CI], p = 0·06), chromosome 13 loss (HR 0·24, 0·07–0·80 [95%CI], p = 0·02), and male sex (HR 1·70, 0·98–2·96 [95% CI], p = 0·06) were incorporated in our Cox model (Fig. 3a). Importantly, histology, *MYC(N)* amplification and extent of resection showed no prognostic utility and were not selected.

We compared the performance of the Cox model in our cohort to its performance in an external validation cohort. The Cox model had a high, bias-corrected c-index (0·72) and showed good calibration (Fig. 3b) in our discovery cohort, however, the model performed poorly when tested against the external validation cohort, with a bias-corrected c-index of 0·60 and poor calibration (Fig. 3c).

We therefore next developed a stratification scheme by selecting well defined molecular disease features [26] from our Cox model, remaining as faithful as possible to the original model, whilst minimising the number of variables upon which risk could be stratified accurately. Combinations of markers were used to categorise patients into risk groups to develop a novel, clinically-deliverable MB_Grp4_ risk-stratification scheme, from established disease cut-offs for projected 5-year PFS [29]. WCA status was retained over chromosome 13 loss, and subgroup 5 was not included as it represents a small proportion of MB_Grp4_.

**Fig. 4:**
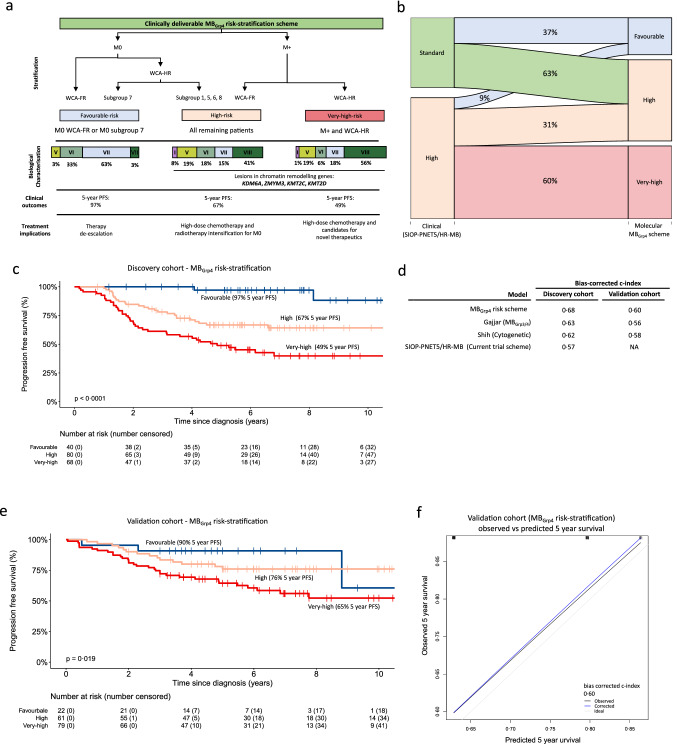
Refined MB_Gpr4_ risk-stratification. **a** Summary of MB_Grp4_ risk-stratification scheme with corresponding biological annotation and clinical implications. **b** Reclassification of risk groups. Sankey plot showing the relationship between the current clinical schemes (SIOP-PNET5-MB [21] [NCT02066220] and SIOP-HR-MB [2] [EudraCT: 2018-004250-17]) and the MB_Grp4_ risk-stratification scheme. **c** Kaplan–Meier plot of PFS by MB_Grp4_ risk-stratification group. At-risk tables are shown in two-year increments with number of patients censored in parentheses and significance shown by p value generated from log-rank test. **d** Performance of MB_Grp4_ risk-stratification scheme in comparison to the current clinical-trial risk scheme (SIOP-PNET5-MB [21] and SIOP-HR-MB [2]), a previously reported cytogenetic risk scheme (Shih et al. [35]) and a published combined MB_Grp3/4_ risk scheme (Gajjar et al. [14]) measured by bias-corrected c-index at 5 years within discovery and validation cohorts. **e** Kaplan–Meier PFS plot for the MB_Grp4_ risk-stratification scheme within the external validation cohort (Northcott et al. [23]). **f** Calibration plot of the MB_Grp4_ risk-stratification within the validation cohort for survival at 5 years alongside the bias-corrected c-index. Abbreviations: *M0* non-metastatic disease, *M +*  metastatic disease, *WCA-FR* whole chromosome aberrations-favourable risk, *WCA-HR* whole chromosome aberrations-high risk, *PFS* progression-free survival

Our refined MB_Grp4_ risk-stratification scheme integrates metastatic stage with both subgroup and WCA status (Fig. 4a), to balance the considerations of predictive accuracy with clinically actionable/practical risk-redistribution, whilst ensuring biological homogeneity within risk-groups. Favourable-risk group membership was defined by non-metastatic patients who are WCA-FR or subgroup 7 (40/188 [21%] of MB_Grp4_, 97% 5-year PFS). Membership of the very-high-risk group was defined by metastatic WCA-HR patients (68/188 [36%], 49% 5-year PFS). Remaining patients (M + patients with WCA-FR, and all M0 patients whose tumours are (i) WCA-HR and (ii) not subgroup 7) displayed a high-risk profile (80/188 [43%], 67% 5-year PFS; Fig. 4c). This scheme performed equivalently (bias-corrected c-index; 0·68) in comparison to the Cox model (bias-corrected c-index; 0·72). Importantly, this scheme returns a favourable-risk group of significant size (21% of MB_Grp4_), with excellent survival outcomes (97% 5-year PFS).

Our MB_Grp4_ risk-stratification scheme thus redefined risk within MB_Grp4_ disease (Fig. 4b); clinical standard-risk (37% of MB_Grp4_) was effectively eliminated and redistributed to favourable (37% of clinical standard-risk) or high-risk (63% of clinical standard-risk) groups. A significant subset of the clinical high-risk group (63% of MB_Grp4_) redistributed to favourable (9% of clinical high-risk) or very-high-risk (60% of clinical high-risk) groups.

To assess the predictive value of our MB_Grp4_ risk-stratification scheme against published and/or clinically utilised stratification models, we again compared performance at 5 years post-diagnosis (Fig. 4d). Our MB_Grp4_ stratification scheme (bias-corrected c-index 0·68) outperformed the scheme in current clinical use (‘current’, bias-corrected c-index 0·57), for the SIOP-HR-MB [EudraCT Number: 2018-004250-17] and SIOP-PNET5-MB [NCT02066220] clinical trials [2, 21]. Furthermore, it performed better against a cytogenetic model (‘cytogenetic’, bias-corrected c-index 0·62) proffered by Shih *et al*. [35], as well as a combined MB_Grp3/4_ scheme proposed by Gajjar *et al.* [15] (‘MB_Grp3/4_’, bias-corrected c-index 0·63). Additionally, the risk-scheme showed good calibration within the discovery cohort (Supplementary Fig. S7, online resource).

Finally, our MB_Grp4_ risk-stratification scheme was reproducible in the independent external validation cohort (Fig. 4e). The performance of the MB_Grp4_ risk-stratification scheme (bias corrected c-index: 0·6) was again better than published and/or clinically utilised stratification models and, unlike the Cox model, calibrated well within the validation cohort (Fig. 4d, f, Supplementary Fig. S8a, b, online resource).

## Discussion

Our interrogation of the specific molecular pathology of ~ 1000 MB_Grp4_ tumours strongly confirms the well-established prognostic importance of metastatic disease within this disease group. We have identified clinically-actionable biological heterogeneity, centred on WCA and methylation subgroups, improving upon initial studies of their molecular and prognostic relevance, which were limited by either cohort size or lack of combined clinico-molecular annotation. The novel integration of these features resolves biologically homogeneous risk groups which allow us to derive a risk-stratification model that reassigns risk-status for 80% of paediatric MB_Grp4_, which outperforms previous schemes (clinical [2, 21], cytogenetic [35] and MB_Grp3/ Grp4_ [15]) and validates in an independent cohort. Importantly, our findings reject previously established disease-wide risk-features (LCA histology and *MYC*(*N*) amplification), showing they have little prognostic relevance in this disease context.

Our MB_Grp4_ risk-stratification scheme balances the considerations of predictive accuracy with clinically actionable and practical risk-redistribution; i.e. the establishment of three meaningfully-sized risk groups. MB_WNT_ represents around 10% of all MB patients and, to date, is the only clinically-actioned favourable-risk group in the disease with CSI dose de-escalation from 24 to 18 Gy [21]. Therefore, our identification of a reproducible favourable-risk group in MB_Grp4_ with excellent outcomes (20% of MB_Grp4_, equating to 8% of all MB with a 97% 5-year PFS) almost doubles the proportion of all MBs suitable for therapy de-escalation approaches.

Our model eliminates clinically-defined standard-risk disease within MB_Grp4_, representing a significant advancement for the future clinical management of MB_Grp4_. Our model redistributes a significant majority (63%) of these patients to a high-risk disease group, potentially explaining why dose reduction strategies within clinically-defined standard-risk MB_Grp4_ has shown inferior survival outcomes in previous trials [20]. The concept of treatment intensification for patients, previously defined on clinical grounds as SR, is currently a matter of careful consideration within the field. Some intensification of chemotherapy or radiotherapy might be appropriate, however for the latter it is conceivable that doses lower than 36 Gy may be sufficient to induce a survival benefit. In clinically-defined high-risk MB_Grp4_ patients from the PNET HR + 5 trial (mostly metastatic), high-dose thiotepa conferred a significant survival advantage [10]. Further work is needed to assess chemotherapy intensification and will be addressed in the SIOP-HR-MB trial [2].

Despite our comprehensive assessment of MB_Grp4_ molecular pathology, significant survival heterogeneity remains within the high and very-high-risk groups; further biomarkers remain to be identified. Whilst outside the scope of this study, future molecular profiling of the transcriptome and proteome is urgently required to identify novel actionable biological pathways in MB_Grp4_. For example, we observed an enrichment of lesions in genes that have coalescing functions as modifiers of H3K4 and H3K27-methylation within a subset of high-risk MB_Grp4_. Mutations in these chromatin modelling genes have been shown to induce aberrant expression patterns of their target histone markers and are associated with worse survival outcomes within non-WNT/non-SHH disease [9]. These epigenetic markers have previously been associated with a radiation-resistant phenotype in experimental systems; therapeutic targeting through BET inhibitors in high-risk non-WNT/non-SHH models has been shown to restore radiation sensitivity and may therefore present a potential novel therapeutic intervention [13].

Our analysis was based on retrospective clinical cohorts and heterogeneous treatment protocols. However, the inclusion of patient data from contemporary cohorts (i.e. HIT-SIOP-PNET4, PNET HR + 5) and a discovery cohort of unprecedented size mitigates this limitation. Furthermore, multimodal therapy in non-infants has become standardised in the last three decades (surgical resection, CSI with adjuvant chemotherapy), legitimising the combination of retrospective cohorts for the development of survival models. The clinical implementation of our MB_Grp4_ risk-stratification scheme is both attractive, feasible and immediately adoptable into clinical studies, given that patients can be mapped using only metastatic status and molecular data routinely collected via DNA methylation array (WCAs/molecular subgroup), which is, increasingly, becoming standard of care in developed countries [8, 19]. Profiling using the Illumina 450k/EPIC DNA methylation arrays has been proven to be robust for detecting copy number variation, in particular WCAs [18].

Our findings refine our understanding of the clinical behaviour of MB_Grp4_ and now require urgent assessment in prospective clinical trials, as a basis for improved diagnostics, personalised therapies and risk-adapted therapeutic strategies.

## Supplementary Information

Below is the link to the electronic supplementary material.

Supplementary file 1 (PDF 2999 KB)

## References

[1] Albright AL, Wisoff JH, Zeltzer PM, Boyett JM, Rorke LB, Stanley P (1996). Effects of medulloblastoma resections on outcome in children: a report from the Children's Cancer Group. Neurosurgery.

[2] Bailey S, André N, Gandola L, Massimino M, Rutkowski S, Clifford SC (2022). Clinical trials in high-risk medulloblastoma: evolution of the SIOP-Europe HR-MB trial. Cancers (Basel).

[3] Cavalli FMG, Remke M, Rampasek L, Peacock J, Shih DJH, Luu B, Garzia L, Torchia J, Nor C, Morrissy AS (2017). Intertumoral heterogeneity within medulloblastoma subgroups. Cancer Cell.

[4] Chang CH, Housepian EM, Herbert C (1969). An operative staging system and a megavoltage radiotherapeutic technic for cerebellar medulloblastomas. Radiology.

[5] Chen Z, Ioris RM, Richardson S, Van Ess AN, Vendrell I, Kessler BM, Buffa FM, Busino L, Clifford SC, Bullock AN, et al (2022) Disease-associated KBTBD4 mutations in medulloblastoma elicit neomorphic ubiquitylation activity to promote CoREST degradation. Cell Death Differ: Doi 10.1038/s41418-022-00983-4 10.1038/s41418-022-00983-4 PMC9525703 35379950

[6] Chevignard M, Câmara-Costa H, Doz F, Dellatolas G (2017). Core deficits and quality of survival after childhood medulloblastoma: a review. Neurooncol Pract.

[7] Clifford SC, Lannering B, Schwalbe EC, Hicks D, O'Toole K, Nicholson SL, Goschzik T, Zur Muhlen A, Figarella-Branger D, Doz F (2015). Biomarker-driven stratification of disease-risk in non-metastatic medulloblastoma: results from the multi-center HIT-SIOP-PNET4 clinical trial. Oncotarget.

[8] Crosier S, Hicks D, Schwalbe EC, Williamson D, Leigh Nicholson S, Smith A, Lindsey JC, Michalski A, Pizer B, Bailey S (2021). Advanced molecular pathology for rare tumours: A national feasibility study and model for centralised medulloblastoma diagnostics. Neuropathol Appl Neurobiol.

[9] Dubuc AM, Remke M, Korshunov A, Northcott PA, Zhan SH, Mendez-Lago M, Kool M, Jones DT, Unterberger A, Morrissy AS (2013). Aberrant patterns of H3K4 and H3K27 histone lysine methylation occur across subgroups in medulloblastoma. Acta Neuropathol.

[10] Dufour C, Foulon S, Geoffray A, Masliah-Planchon J, Figarella-Branger D, Bernier-Chastagner V, Padovani L, Guerrini-Rousseau L, Faure-Conter C, Icher C (2021). Prognostic relevance of clinical and molecular risk factors in children with high-risk medulloblastoma treated in the phase II trial PNET HR+5. Neuro Oncol.

[11] Ellison DW, Kocak M, Dalton J, Megahed H, Lusher ME, Ryan SL, Zhao W, Nicholson SL, Taylor RE, Bailey S (2011). Definition of disease-risk stratification groups in childhood medulloblastoma using combined clinical, pathologic, and molecular variables. J Clin Oncol.

[12] Ellison DW, Onilude OE, Lindsey JC, Lusher ME, Weston CL, Taylor RE, Pearson AD, Clifford SC, United Kingdom Children's Cancer Study Group Brain Tumour C (2005). beta-Catenin status predicts a favorable outcome in childhood medulloblastoma: the United Kingdom Children's Cancer Study Group Brain Tumour Committee. J Clin Oncol.

[13] Gabriel NN, Balaji K, Jayachandran K, Inkman M, Zhang J, Dahiya S, Goldstein M (2022). Loss of H3K27 trimethylation promotes radiotherapy resistance in medulloblastoma and induces an actionable vulnerability to BET inhibition. Cancer Res.

[14] Gajjar A, Robinson GW, Smith KS, Lin T, Merchant TE, Chintagumpala M, Mahajan A, Su J, Bouffet E, Bartels U, et al Outcomes by Clinical and Molecular Features in Children With Medulloblastoma Treated With Risk-Adapted Therapy: Results of an International Phase III Trial (SJMB03). Journal of Clinical Oncology Doi: 10.1200/jco.20.01372 10.1200/JCO.20.01372 PMC10166353 33405951

[15] Gajjar A, Robinson GW, Smith KS, Lin T, Merchant TE, Chintagumpala M, Mahajan A, Su J, Bouffet E, Bartels U (2021). Outcomes by clinical and molecular features in children with medulloblastoma treated with risk-adapted therapy: results of an international phase iii trial (SJMB03). J Clin Oncol.

[16] Goschzik T, Schwalbe EC, Hicks D, Smith A, Zur Muehlen A, Figarella-Branger D, Doz F, Rutkowski S, Lannering B, Pietsch T (2018). Prognostic effect of whole chromosomal aberration signatures in standard-risk, non-WNT/non-SHH medulloblastoma: a retrospective, molecular analysis of the HIT-SIOP PNET 4 trial. Lancet Oncol.

[17] Hill RM, Kuijper S, Lindsey JC, Petrie K, Schwalbe EC, Barker K, Boult JKR, Williamson D, Ahmad Z, Hallsworth A (2015). Combined MYC and P53 defects emerge at medulloblastoma relapse and define rapidly progressive, therapeutically targetable disease. Cancer Cell.

[18] Hovestadt V, Zapatka M (2015) conumee: Enhanced copy-number variation analysis using Illumina DNA methylation arrays. R package version 1.9.0. http://bioconductor.org/packages/conumee/. 10.1093/bioinformatics/btae029 PMC10868300 38244574

[19] Louis DN, Perry A, Wesseling P, Brat DJ, Cree IA, Figarella-Branger D, Hawkins C, Ng HK, Pfister SM, Reifenberger G (2021). The 2021 WHO classification of tumors of the central nervous system: a summary. Neuro Oncol.

[20] Michalski JM, Janss AJ, Vezina LG, Smith KS, Billups CA, Burger PC, Embry LM, Cullen PL, Hardy KK, Pomeroy SL (2021). Children's oncology group phase iii trial of reduced-dose and reduced-volume radiotherapy with chemotherapy for newly diagnosed average-risk medulloblastoma. J Clin Oncol.

[21] Mynarek M, Milde T, Padovani L, Janssens GO, Kwiecien R, Mosseri V, Clifford SC, Doz F, Rutkowski S (2021). SIOP PNET5 MB trial: history and concept of a molecularly stratified clinical trial of risk-adapted therapies for standard-risk medulloblastoma. Cancers (Basel).

[22] Mynarek M, Obrecht D, Sill M, Sturm D, Kloth-Stachnau K, Selt F, Ecker J, von Hoff K, Juhnke B-O, Goschzik T (2023). Identification of low and very high-risk patients with non-WNT/non-SHH medulloblastoma by improved clinico-molecular stratification of the HIT2000 and I-HIT-MED cohorts. Acta Neuropathol.

[23] Northcott PA, Buchhalter I, Morrissy AS, Hovestadt V, Weischenfeldt J, Ehrenberger T, Gröbner S, Segura-Wang M, Zichner T, Rudneva VA (2017). The whole-genome landscape of medulloblastoma subtypes. Nature.

[24] Northcott PA, Korshunov A, Witt H, Hielscher T, Eberhart CG, Mack S, Bouffet E, Clifford SC, Hawkins CE, French P (2011). Medulloblastoma comprises four distinct molecular variants. J Clin Oncol.

[25] Northcott PA, Lee C, Zichner T, Stütz AM, Erkek S, Kawauchi D, Shih DJ, Hovestadt V, Zapatka M, Sturm D (2014). Enhancer hijacking activates GFI1 family oncogenes in medulloblastoma. Nature.

[26] Northcott PA, Robinson GW, Kratz CP, Mabbott DJ, Pomeroy SL, Clifford SC, Rutkowski S, Ellison DW, Malkin D, Taylor MD (2019). Medulloblastoma Nature reviews. Dis Primers.

[27] Northcott PA, Shih DJ, Peacock J, Garzia L, Morrissy AS, Zichner T, Stutz AM, Korshunov A, Reimand J, Schumacher SE (2012). Subgroup-specific structural variation across 1,000 medulloblastoma genomes. Nature.

[28] Pietsch T, Schmidt R, Remke M, Korshunov A, Hovestadt V, Jones DT, Felsberg J, Kaulich K, Goschzik T, Kool M (2014). Prognostic significance of clinical, histopathological, and molecular characteristics of medulloblastomas in the prospective HIT2000 multicenter clinical trial cohort. Acta Neuropathol.

[29] Ramaswamy V, Remke M, Bouffet E, Bailey S, Clifford SC, Doz F, Kool M, Dufour C, Vassal G, Milde T (2016). Risk stratification of childhood medulloblastoma in the molecular era: the current consensus. Acta Neuropathol.

[30] Richardson S, Hill RM, Kui C, Lindsey JC, Grabovksa Y, Keeling C, Pease L, Bashton M, Crosier S, Vinci M (2021). Emergence and maintenance of actionable genetic drivers at medulloblastoma relapse. Neuro Oncol.

[31] Ryan SL, Schwalbe EC, Cole M, Lu Y, Lusher ME, Megahed H, O'Toole K, Nicholson SL, Bognar L, Garami M (2012). MYC family amplification and clinical risk-factors interact to predict an extremely poor prognosis in childhood medulloblastoma. Acta Neuropathol.

[32] Schwalbe EC, Hicks D, Rafiee G, Bashton M, Gohlke H, Enshaei A, Potluri S, Matthiesen J, Mather M, Taleongpong P (2017). Minimal methylation classifier (MIMIC): a novel method for derivation and rapid diagnostic detection of disease-associated DNA methylation signatures. Sci Rep.

[33] Schwalbe EC, Lindsey JC, Nakjang S, Crosier S, Smith AJ, Hicks D, Rafiee G, Hill RM, Iliasova A, Stone T (2017). Novel molecular subgroups for clinical classification and outcome prediction in childhood medulloblastoma: a cohort study. Lancet Oncol.

[34] Sharma T, Schwalbe EC, Williamson D, Sill M, Hovestadt V, Mynarek M, Rutkowski S, Robinson GW, Gajjar A, Cavalli F (2019). Second-generation molecular subgrouping of medulloblastoma: an international meta-analysis of Group 3 and Group 4 subtypes. Acta Neuropathol.

[35] Shih DJ, Northcott PA, Remke M, Korshunov A, Ramaswamy V, Kool M, Luu B, Yao Y, Wang X, Dubuc AM (2014). Cytogenetic prognostication within medulloblastoma subgroups. J Clin Oncol.

[36] von Bueren AO, Kortmann RD, von Hoff K, Friedrich C, Mynarek M, Muller K, Goschzik T, Zur Muhlen A, Gerber N, Warmuth-Metz M (2016). Treatment of children and adolescents with metastatic medulloblastoma and prognostic relevance of clinical and biologic parameters. J Clin Oncol.

[37] Williamson D, Schwalbe EC, Hicks D, Aldinger KA, Lindsey JC, Crosier S, Richardson S, Goddard J, Hill RM, Castle J (2022). Medulloblastoma group 3 and 4 tumors comprise a clinically and biologically significant expression continuum reflecting human cerebellar development. Cell Rep.

[38] Zhukova N, Ramaswamy V, Remke M, Pfaff E, Shih DJ, Martin DC, Castelo-Branco P, Baskin B, Ray PN, Bouffet E (2013). Subgroup-specific prognostic implications of TP53 mutation in medulloblastoma. J Clin Oncol.

