# Supplementary material for: Molecular characterisation defines clinically-actionable heterogeneity within Group 4 medulloblastoma and improves disease risk-stratification
Source: Acta Neuropathol. 2023 Apr 4;145(5):651–66. doi: 10.1007/s00401-023-02566-0 (PMC10119222; doi:10.1007/s00401-023-02566-0)
Supplement: Supplementary file 1 — (PDF 2999 KB) [file 401_2023_2566_MOESM1_ESM.pdf]

Molecular characterisation defines clinically-actionable heterogeneity within Group 4 medulloblastoma and improves disease risk-stratification

Goddard et al.

Supplementary Material

Supplementary Methods

Methylation subgrouping

Tumour samples with available Illumina HumanMethylation450K/EPIC array were classified if the confidence score exceeded 0·7 and were assigned to the four consensus subgroups using established techniques [14]. Only those confidently assigned MB_{Grp4} tumours were included in the discovery cohort. Our cohorts were checked for potential duplication by examining pairwise correlations of 65 genotyping probes for samples with methylation array [14]. Second-generation methylation subtypes were assigned using the DNA methylation classifier (www.molecularneuropathology.org/mnp) (confidence score ≥0·8) [2]. Unsupervised t-SNE analysis of the 10000 most variable methylated probes was used to visualise subgroup classifications including non-classified (NC) samples, using the R package 'Rtsne' v0.16 (the following non-default parameters were used: theta=0, perplexity=29, max_iter=10000). IDATs were processed as previously described [6].

Samples from HIT-SIOP-PNET4 and those with DNA derivatives that were poor quality and/or low concentration and unsuitable for Illumina HumanMethylation450K/EPIC analysis were classified using the minimal methylation classifier (MIMIC), detailed previously [5, 13].

Copy Number Analysis

Chromosome arm-level copy number estimates were derived from Illumina HumanMethylation 450K/EPIC array data, using the package 'Conumee' (R/Bioconductor) v1.16.0 as previously described [14]; the conumee annotation function was modified to use the IlluminaHumanMethylationEPIC "ilm10b4.hg19" manifest. Molecular inversion probe (MIP) array was used for the HIT-SIOP-PNET4 cohort to call arm-level copy number as previously described [5].

Focal copy number variants (CNVs) were identified from samples with DNA methylation array in our sequencing cohort (n=171/172) using previously identified thresholds [12] from probe intensity values located within the genomic location of annotated genes. Samples with a 'conumee' noise score >2 were not assigned focal CNVs and all calls were manually verified. Arm-level and focal CNVs of validation samples was performed using these methods.

Next Generation Sequencing

Targeted gene panel (n=168) and whole-exome (n=4) sequencing was carried out to interrogate the mutational status of those genes previously reported as frequently mutated putative driver genes in MB (n=172). Mutational analysis of exomes was restricted to genes shared between the panel and exome sequencing data (Supplementary Table 2). We used the Agilent SureSelectXT Low Input system library preparation protocol with subsequent sequencing using the Illumina HiSeq2500.

Next generation sequencing (NGS) datasets were analysed for coding/exonic region variants using Genome Analysis Toolkit (GATK) version 3.7, according to Broad Institute's best practices. Exomes were analysed as previously described [12]. Targeted gene panels were analysed similarly however GATK Hard-filtering was used instead of VQSR. Sequences were aligned against the GRCh37/Hg19 human reference genome. Variants were predicted pathogenic if their consequence included coding or splice donor/acceptor mutations, max allele frequency <0·01 (ExAC, GnomAD/exomes, 1000Genomes, ALFA) and were predicted to be deleterious by both CAROL and FATHMM prediction tools. Mutations in the TERT promotor were also included [7]. Variants with COSMIC (Catalogue of Somatic Mutations in Cancer) annotations flagged as SNPs were subsequently removed.

RNA-sequencing data was generated as previously described [14]. Transcriptomic analysis was used to establish *PRDM6* and *GFI1/1B* overexpression for samples within the sequencing cohort (83/172) from established techniques [9, 10].

Mutational and overexpression calls for validation samples were obtained from the corresponding study supplementary material [9].

Statistical and survival analysis

For the re-derivation of WCA groups, we considered WCAs with a frequency ≥15% which were assessed in univariable analysis using Cox regression. Combinations of WCAs were assessed using methods detailed previously [5]. To assess the biological relationships between WCAs, we calculated a distance matrix using the binary distance measure within the R function 'dist' of all WCAs. We performed hierarchical clustering using average linkage with the function 'hclust' and visualised the resulting dendrogram and associated clinico-molecular information using the 'aheatmap' function in the 'NMF' package v0.24.0. Fisher's exact tests were used to evaluate mutual exclusivity and co-occurrence of recurrent WCA (≥10%), significant associations were defined by p value <0·05 adjusted for multiple testing using the Benjamini-Hochberg procedure.

We examined survival relationships of features with a frequency ≥10% using univariable Cox analysis within MB_{Grp4} methylation and WCA subgroups, with the exception of subgroup 1 (n=9).

For the MB_{Grp4} survival cohort (n=326), we assessed all novel clinico-molecular variables present at a frequency ≥10% alongside established features and treatment variables using univariable Cox analysis. Multivariable Cox analysis was carried out using samples with an existing second-generation methylation subgroup call and available survival data (n=213; 80 events). We assessed missing data within these samples and used imputation to generate a complete dataset using the 'aregImpute' function from the R package 'Hmisc' v4.7-0 for the following variables: metastatic stage, large-cell/anaplastic histology, subtotal resection, *MYC/MYCN* amplification and dose of craniospinal irradiation. Dose of chemotherapy was not imputed as there was ≥20% missing data and therefore was not considered in multivariable analysis.

Whilst striving to balance the number of observed events in our cohort with the number of variables to construct a model, we employed backwards model selection of variables using the 'stepAIC' function from the R package 'MASS', v7.3-53.1. We considered variables from established disease features (metastatic disease, extent of resection, LCA pathology, *MYCN* amplification, i17q, sex and dose of CSI) alongside biologically and clinically significant molecular factors (WCA status, subgroup 7, chromosome 13 loss, subgroup 5, chromosome 18 gain). Proportionality of hazards was assessed using the 'cox.zph' function in the R package 'survival' v3.2-7.

A clinically deliverable MB_{Grp4} risk-stratification scheme was generated from combinations of markers by categorising patients using selected variables into risk groups with established disease cut-offs for projected 5-year PFS: favourable-risk ≥90% survival, standard-risk 75–90% survival, high-risk 50–75% survival and very high risk, <50% survival [11].

The calibration and discrimination of the Cox models and derived risk schemes were tested on both discovery and validation cohorts using the R package 'rms' v6.3-0. To assess how well model predictions fitted to observed data, *i.e.* the difference between estimated and observed absolute risk, model calibration was performed using the function 'calibrate', with 1000 bootstraps at 5 years after diagnosis. Model discrimination was assessed using the rms function 'validate', once again with 1000 bootstraps and the bias-corrected C-index at 5-years from diagnosis was calculated. Finally, we compared our MB_{Grp4} risk-stratification scheme to current clinical risk-schemes [1, 8] and published molecularly derived schemes [4, 15].

An independent external cohort [9] with available clinico-molecular and PFS data was used for the validation of our multivariable Cox model and risk-stratification scheme. For validation samples, no treatment information was available; analysis was therefore restricted to patients ≥3 years old at diagnosis with complete information on available biological and clinical parameters.

Progression-free survival (PFS) was defined as the time from surgery to first event (progression, relapse or death) or last follow-up. Data was right-censored at last follow-up for patients who had confirmed second malignancies or died of other causes and was not counted as disease progression. Missing data was assumed to be missing completely at random.

Fisher's exact and Chi square tests were used to assess associations between categorical variables. Kruskal-Wallis, Mann-Whitney U, ANOVA and t-tests were used to compare continuous variables between groups. Features with a cohort-wide frequency of ≥5% or with a subtype-specific frequency ≥10% were considered for subgroup analysis. Significant associations were defined as having an adjusted p value of <0·05 using the Benjamini-Hochberg procedure to correct for multiple testing.

Statistical and bioinformatic analyses were done using R v4.0.4.

Supplementary Figure 1

a

Discovery Cohort

	Total (n = 362)	Survival (n = 323)
UK-CCLG + collaborating centres	228	189
PNET HR+5	29	29
SIOP-UKCCSG-PNET3	27	27
HIT-SIOP-PNET4	78	78

Validation Cohort

	Total (n = 668)	Survival (n = 353)
Cavalli *et al*	248	191 (OS)
Northcott *et al*	420	162 (PFS)

b

Cohort	All	Subgroup 1	Subgroup 5	Subgroup 6	Subgroup 7	Subgroup 8	WCA-FR	WCA-HR	Figures
**MB_{Grp4} Discovery Cohort**	362	9	36	38	73	92	105	248	Table 1, Sup Fig. 1, Sup Table 1
**Second generation methylation subgroups**									
Discovery cohort	248	9	36	38	73	92	-	-	Fig. 1a-b, Sup Fig. 4a-d
Validation cohort	602	18	51	85	196	252	-	-	Sup Fig. 4c-d
**WCA subroups**									
Discovery cohort	353	-	-	-	-	-	105	248	Fig. 2a-b, Sup Fig. 3, 6a-b
Validation cohort	668	-	-	-	-	-	156	512	Sup Fig. 6a-b
**Survival Cohorts**									
MB_{Grp4} Discovery survival cohort	323	8	32	34	54	85	94	220	Fig. 3a-b, 4a-d, Sup Fig. 1e, 7
Second generation methylation survival cohort	205	-	32	34	54	85	-	-	Fig. 1c-i, Sup Fig. 4e-f and 5
WCA survival cohort	314	-	-	-	-	-	94	220	Fig. 2c-k, Sup Fig. 2
Validation survival cohort (OS)*	191	-	-	-	-	-	46	145	Sup Fig. 6c
Validation survival cohort (PFS)#	162	6	8	31	41	76	34	127	Fig. 3c, 4d-f, Sup Fig. 8

c

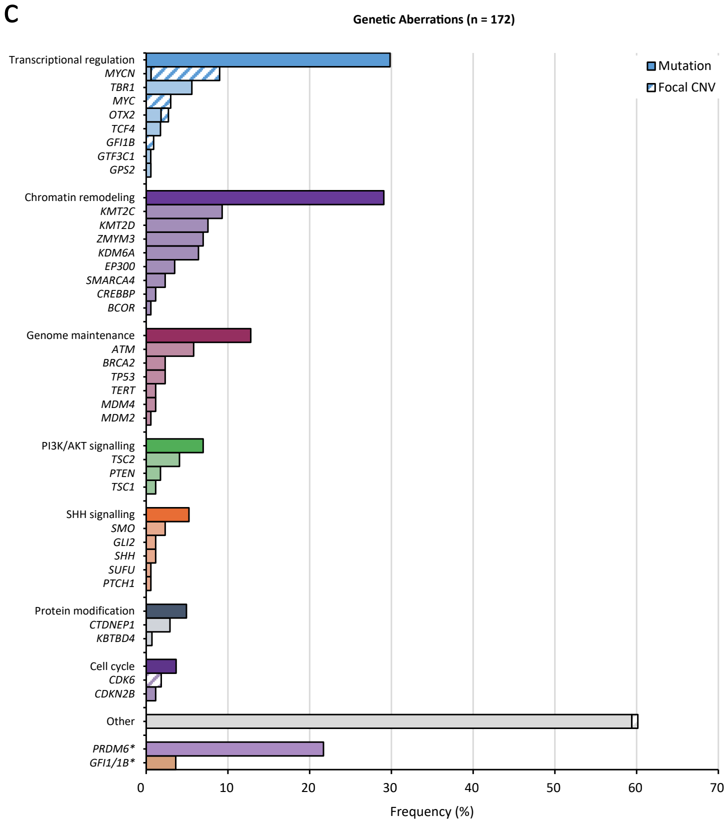

d

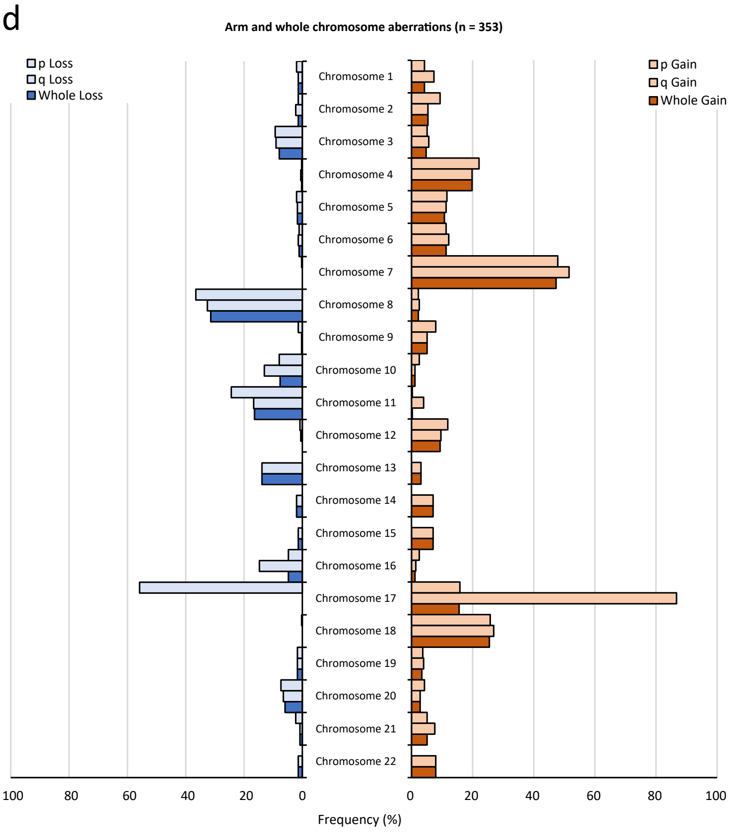

e

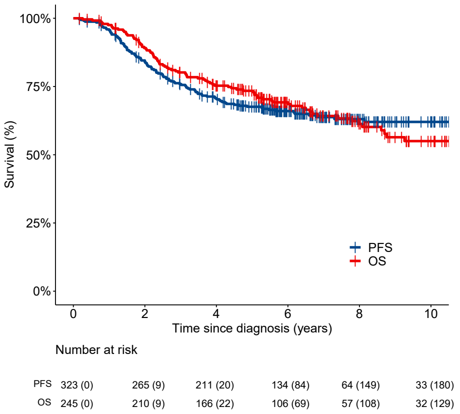

Supplementary Table 1

	WNT (n = 79)	SHH (n = 195)	Grp3 (n = 215)	Grp4 (n = 362)	Grp4 vs non Grp4	Grp4 vs Grp3
**Sex**						
Male (M)	26 (35%)	108 (56%)	161 (75%)	242 (67%)	NS	NS
Female (F)	49 (65%)	86 (44%)	54 (25%)	119 (33%)		
M:F ratio	0·5:1	1·3:1	3:1	2:1		
**Age at Diagnosis (years)**						
Median	10 (4·7-20·3)	6 (0-43)	5 (0-16)	8 (0·2-20)	**0·0002**	**<0·0001**
**Pathology**						
CLA	59 (84%)	54 (33%)	138 (77%)	277 (84%)		
DN/MBEN	2 (3%)	81 (49%)	5 (3%)	30 (9%)	**<0·0001**	**<0·0001**
LCA	9 (13%)	29 (18%)	36 (20%)	21 (6%)		
**Metastatic stage**						
M0	63 (91%)	122 (72%)	112 (57%)	212 (66%)	NS	NS
M+	6 (9%)	48 (28%)	85 (43%)	111 (64%)		
**Resection**						
STR	9 (12%)	33 (19%)	64 (32%)	92 (28%)	NS	NS
GTR	65 (88%)	145 (81%)	133 (68)	242 (72%)		
***MYC* amplification**						
Amplified	1 (1%)	3 (2%)	32 (16%)	6 (2%)	**0·0002**	**<0·0001**
Not amplified	72 (99%)	181 (98%)	168 (84%)	327 (98%)		
***MYCN* amplification**						
Amplified	0	30 (16%)	4 (2%)	24 (7%)	NS	**0·01**
Not amplified	73 (100%)	154 (84%)	195 (98%)	308 (93%)		
**Chromosome 17**						
i17q	1 (2%)	2 (1%)	46 (22%)	196 (56%)	**<0·0001**	**<0·0001**
No i17q	54 (98%)	170 (99%)	166 (78%)	157 (44%)		
**WCA group**						
WCA-FR	-	-	49 (23%)	105 (30%)	NA	NS
WCA-HR	-	-	163 (77%)	248 (70%)		
**Grp3/4 second generation methylation subgroups**						
1	-	-	2 (1%)	9 (4%)		
2	-	-	54 (33%)	0		
3	-	-	48 (29%)	0		
4	-	-	48 (29%)	0	NA	**<0·0001**
5	-	-	10 (6%)	36 (15%)		
6	-	-	0	38 (16%)		
7	-	-	2 (1%)	73 (30%)		
8	-	-	0	92 (38%)		

Supplementary Table 2

Chromatin remodelling	Transcriptional regulation	Genome Maintenance	SHH Signalling	PI3K/ATK	Protein Modification	Cell Cycle	WNT signalling	Other		
KMT2C	*MYCN*	*ATM*	*SMO*	*TSC2*	*CTDNEP1*	*CDK6*	*APC*	*ABCA13*	*FLNA*	*RYR3*
KMT2D	*TBR1*	*BCRA2*	*GLI2*	*PTEN*	*KBTBD4*	*CDKN2A*	*CSNK2*	*ALPK2*	*KIF26B*	*SNCAIP*
KDM6A	*MYC*	*TP53*	*SHH*	*TSC1*	*FBXW7*	*CDKN2B*	*CTNNB1*	*CACNA1D*	*LRP1B*	*SPTB*
ZMYM3	*TCF4*	*TERT*	*SUFU*	*AKT1*				*CBFA2T2*	*LTBP4*	*TNXB*
EP300	*OTX2*	*MDM4*	*PTCH1*	*PI3KCA*				*CDH7*	*MAN2C1*	
SMARCA4	*GTF3C1*	*MDM2*						*DDX3X*	*NBAS*	
CREBBP	*GFI1B*	*PALB2*						*EPPK1*	*NEB*	
BCOR	*LDB1*							*EYA4*	*NLRP5*	
	GPS2							*FCGBP*	*PRKAR1A*	

Supplementary Figure 2

a

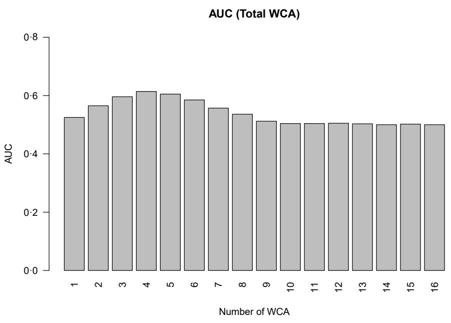

b

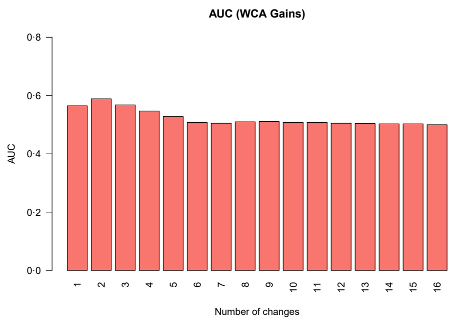

c

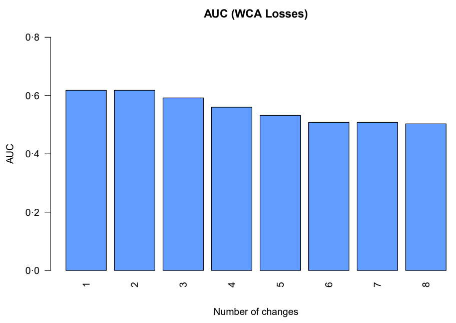

d

Prognostic WCAs	Univariable (n=314)		
	n	HR (95% CI)	p
**Loss 8**	94/314	**0·27 (0·15-0·48)**	**<0·0001**
**Loss 11**	55/314	**0·23 (0·10-0·53)**	**0·0005**
**Gain 7**	152/314	**0·51 (0·35-0·77)**	**0·001**
**Gain 18**	81/314	**0·56 (0·34-0·92)**	**0·02**
**Gain 4**	65/314	0·80 (0·49-1·32)	0·45
**Gain 17**	52/314	0·58 (0·32-1·06)	0·08
**i17q**	174/314	1·11 (0·75-1·63)	0·61

e

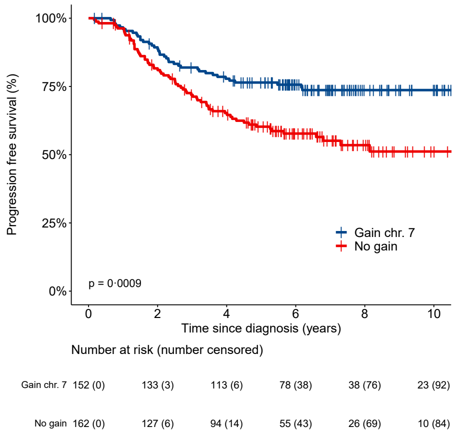

f

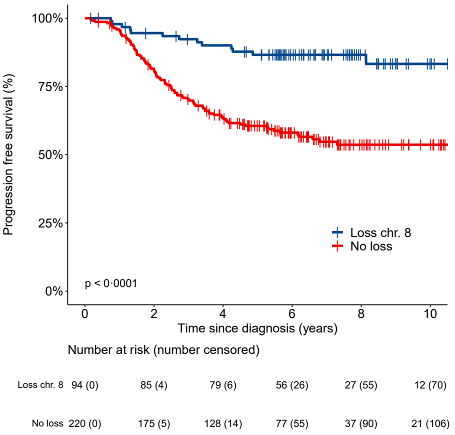

g

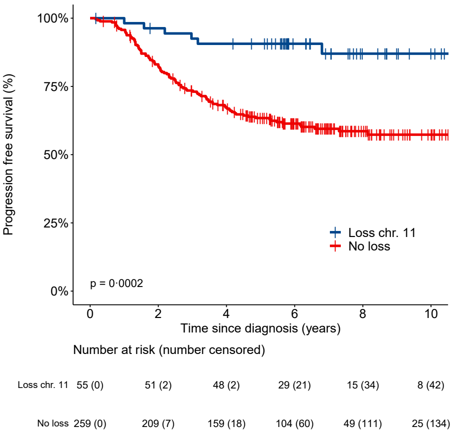

h

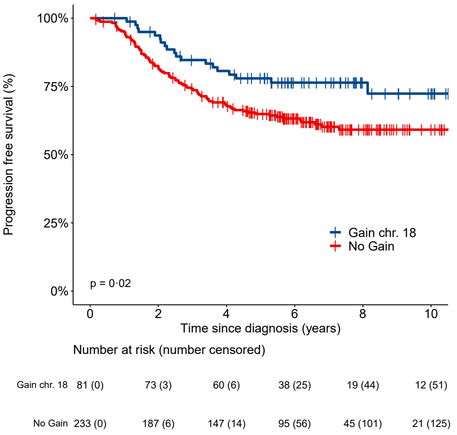

i

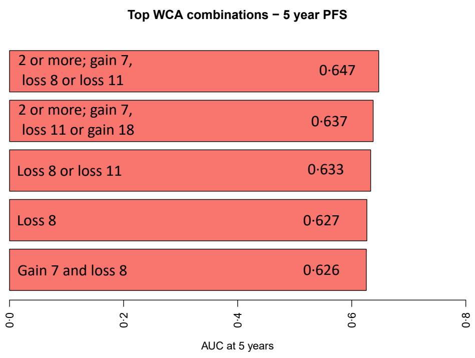

Supplementary Figure 3

a

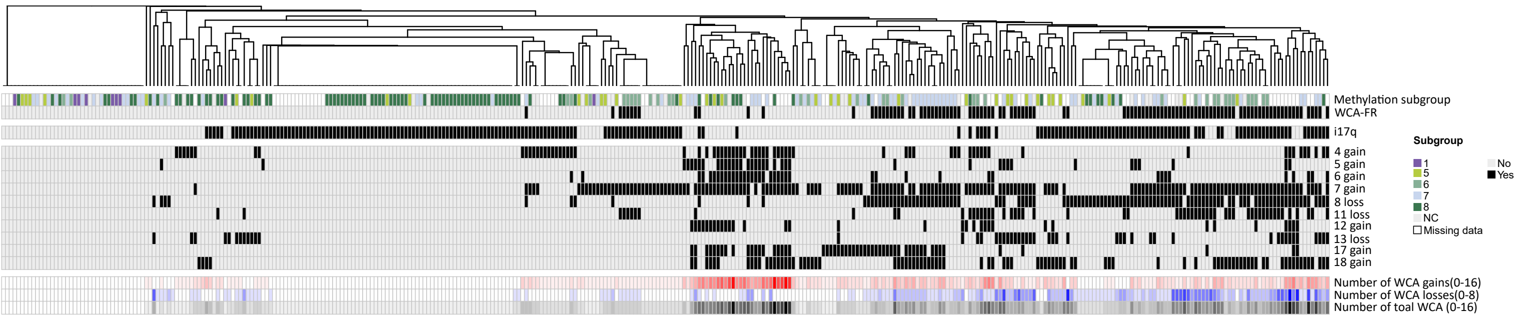

b

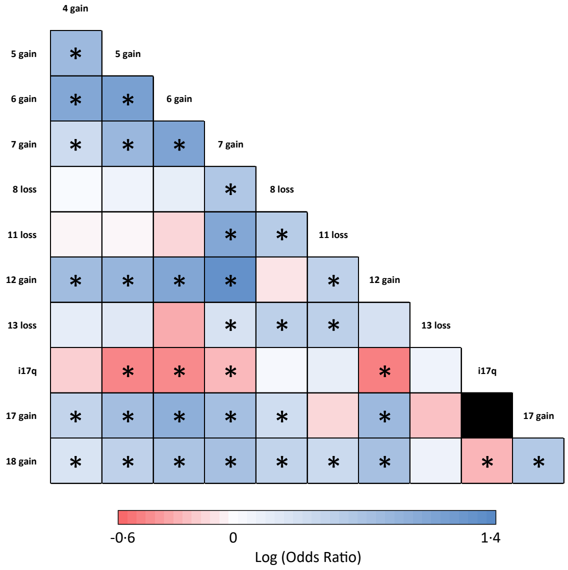

Supplementary Figure 4

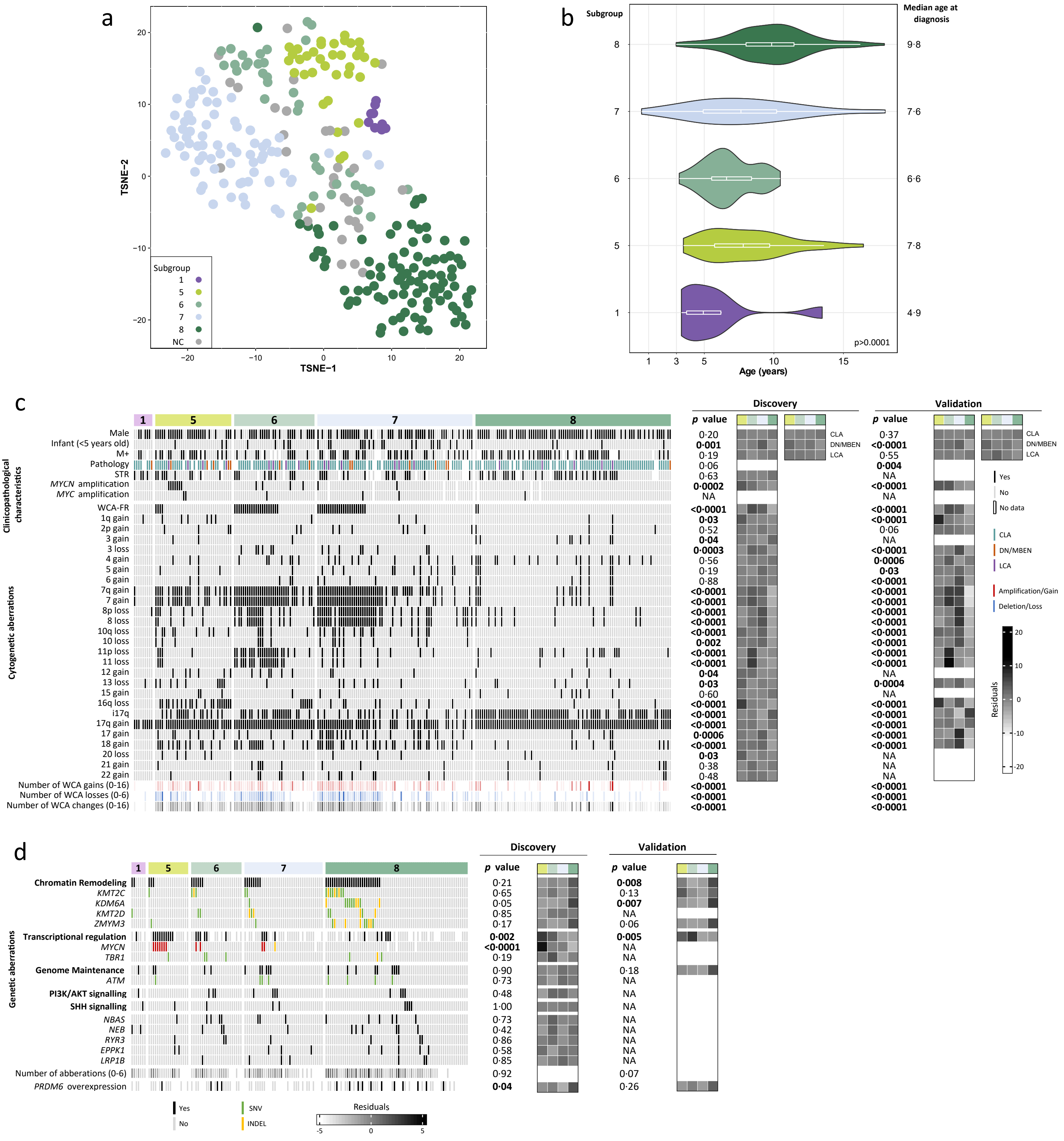

e

Subgroup 5 (n=32)

	Univariable		
	n	HR (95% CI)	p
**Clinicopathological features**			
M+	16/29	1·79 (0·61-5·25)	0·29
STR	9/21	1·30 (0·45-3·77)	0·63
MYCN	7/31	2·06 (0·71-5·98)	0·18
Male	24/32	1·81 (0·52-6·31)	0·35
LCA	3/28	3·18 (0·86-11·80)	0·09
**Molecular features**			
13 loss	7/32	0·12 (0·02-0·94)	**0·04**
i17q	14/32	1·35 (0·52-3·51)	0·54
WCA-FR	4/32	0·28 (0·04-2·13)	0·22
Transcriptional regulation	10/16	0·80 (0·23-2·86)	0·74
Chromatin remodelling	3/19	2·67 (0·69-10·4)	0·16
Genome maintenance	2/19	0·61 (0·08-4·47)	0·63
**Treatment**			
HD CTX	23/31	0·20 (0·02-1·57)	0·13
HD CSI	8/24	0·94 (0·30-2·93)	0·92

f

Subgroup 8 (n=85)

	Univariable		
	n	HR (95% CI)	p
**Clinicopathological features**			
M+	38/85	1·77 (0·95-3·71)	0·13
STR	31/83	0·66 (0·34-1·28)	0·22
Male	65/85	1·25 (0·54-2·64)	0·55
**Molecular features**			
i17q	65/85	0·7 (0·37-1·55)	0·44
Chromatin remodelling	23/63	0·56 (0·25-1·25)	0·16
Genome maintenance	9/63	0·96 (0·37-2·50)	0·93
Transcriptional regulation	8/42	2·14 (0·82-5·58)	0·12
**Treatment**			
CTX	73/84	0·98 (0·41-2·37)	0·97
HD CTX	15/66	0·64 (0·24-1·67)	0·36
HD CSI	63/80	1·18 (0·49-2·85)	0·71

Supplementary Figure 5

a

M0 Subgroup 6 - WCA-FR vs WCA-HR

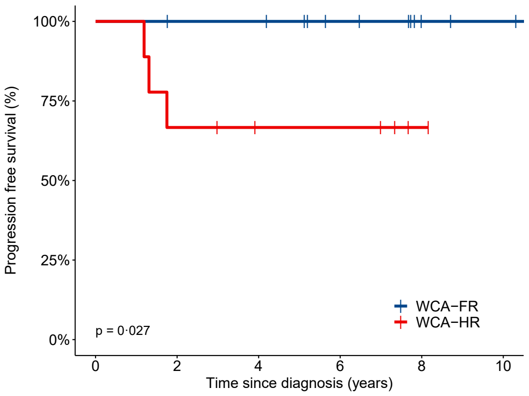

Number at risk (number censored)

WCA−FR	13 (0)	12 (1)	12 (1)	8 (5)	3 (10)	2 (11)
WCA−HR	9 (0)	6 (0)	4 (2)	4 (2)	1 (5)	0 (6)

b

Subgroup 7 - WCA-FR vs WCA-HR

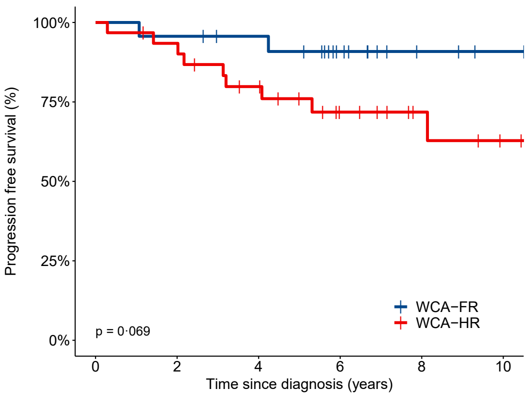

Number at risk (number censored)

WCA−FR	23 (0)	22 (0)	20 (2)	13 (8)	6 (15)	4 (17)
WCA−HR	31 (0)	28 (1)	22 (3)	14 (9)	8 (15)	5 (17)

c

M0 Subgroup 7 - WCA-FR vs WCA-HR

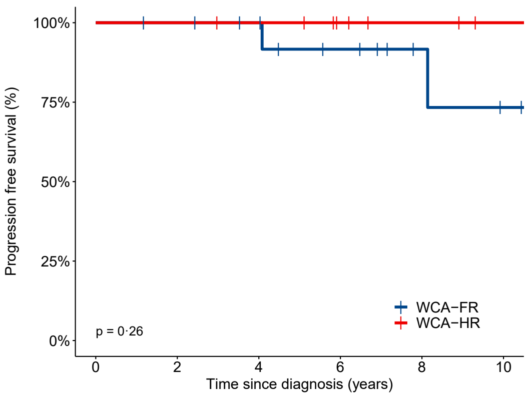

Number at risk (number censored)

WCA−FR	16 (0)	15 (1)	13 (3)	9 (6)	5 (10)	3 (11)
WCA−HR	9 (0)	9 (0)	8 (1)	5 (4)	3 (6)	1 (8)

Supplementary Figure 6

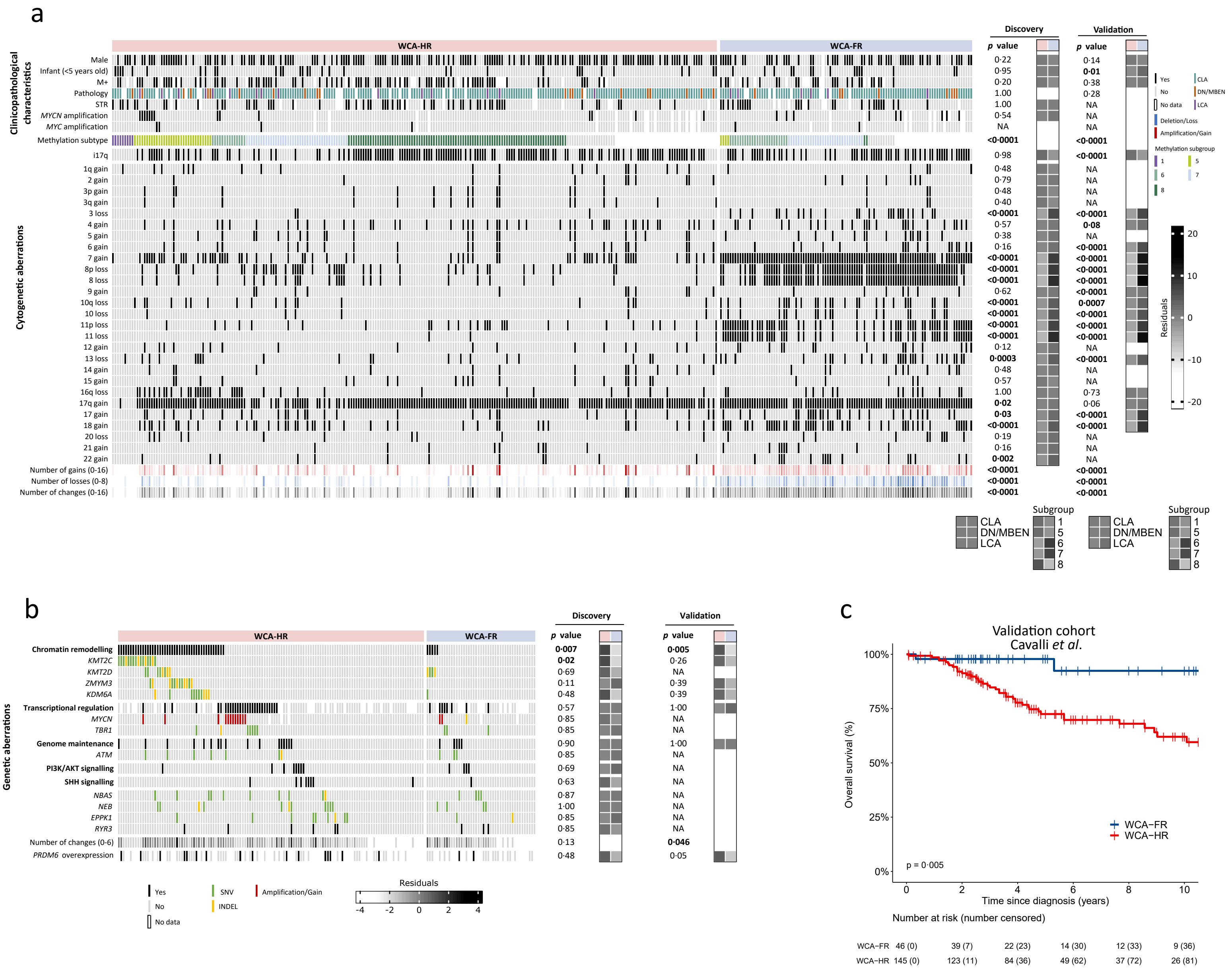

Supplementary Figure 7

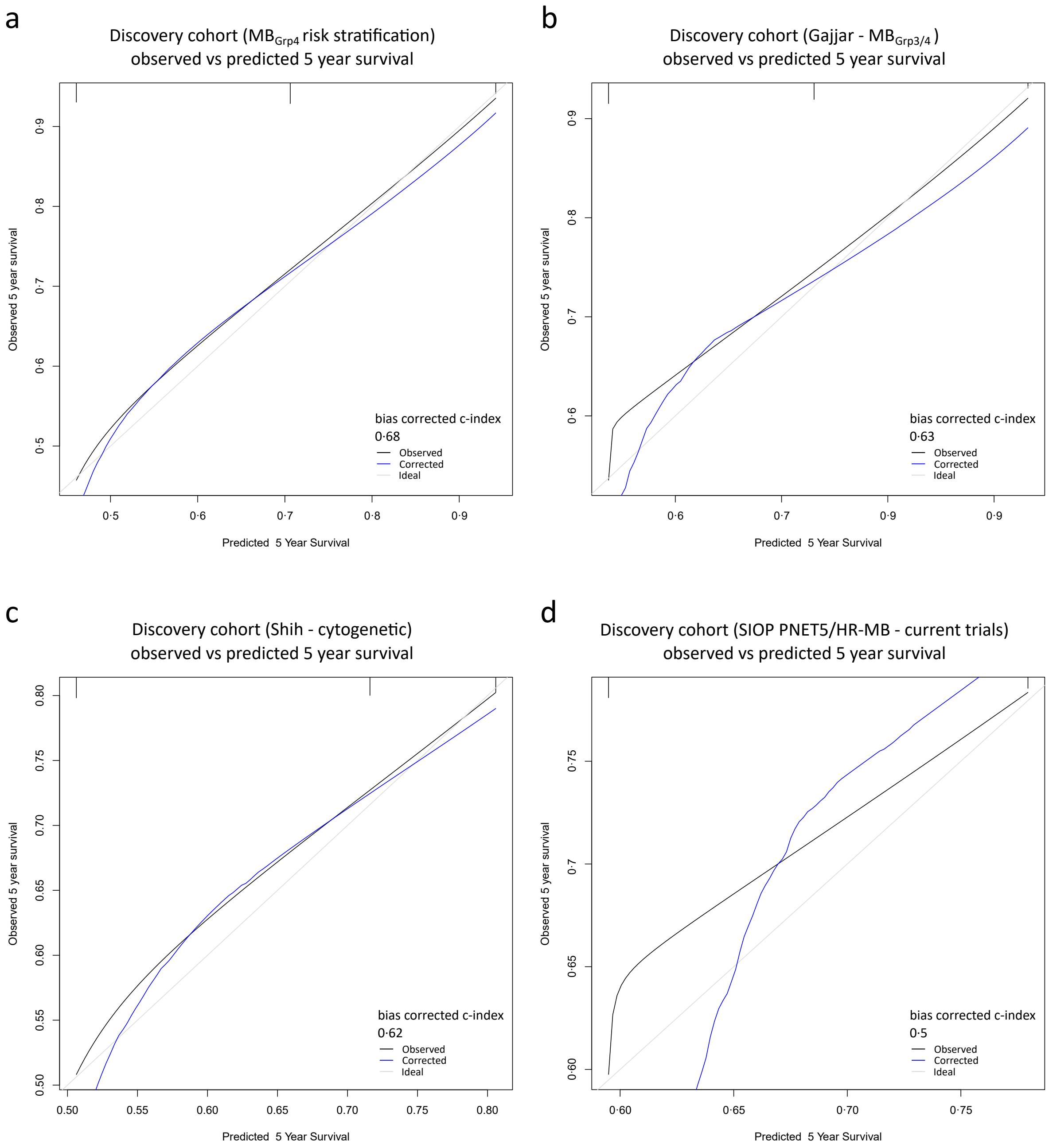

Supplementary Figure 8

a

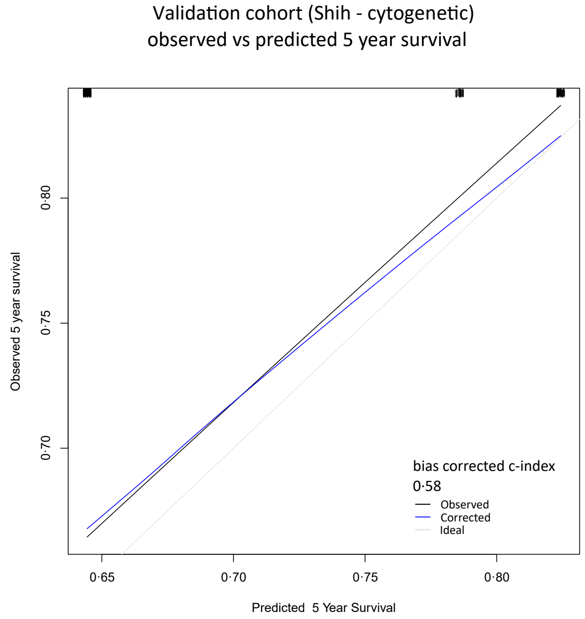

b

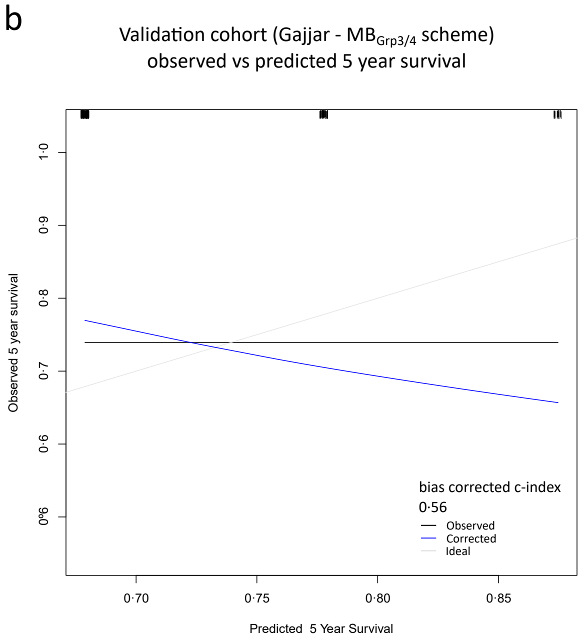

Supplementary Figure Legends

Supplementary Fig. 1: a) Composition of the discovery and validation cohort. The discovery survival cohort is comprised of a subset of patients ≥3 years of age who received craniospinal irradiation-based therapies. The validation cohort consists of cases from Cavalli *et al*. [3] and Northcott *et al*. [9]. b) Composition and use of primary discovery and published cohorts within the study with breakdown of samples used in analysis. For the validation survival cohorts, these were considered separately and consisted of either samples from Cavalli *et al*. [3] * or Northcott *et al*. [9] # due to availability of survival data for only OS or PFS in each cohort, respectively. For validation of clinco-molecular associations, the validation cohort consisted of Cavalli *et al*. [3] and Northcott *et al*. [9] combined. c) Frequencies of genetic aberrations (mutations (solid), focal copy number variations (striped)) in the sequencing cohort (n=172); genes with overlapping functions are shown assembled into biologically-relevant groups. 'Other' represents genes not assigned into biologically-relevant groups. *high gene expression from RNA-seq. d) Recurrent arm-level and whole chromosome gains (red)/losses (blue) for autosomal chromosomes. e) Kaplan-Meier curves for both PFS and OS of MB_{Grp4}. UK-CCLG = UK Children's Cancer and Leukaemia Group, SIOP = The International Society of Pediatric Oncology, UKCCSG = United Kingdom Children's Cancer Study Group, OS = overall survival, PFS = progression-free survival.

Supplementary Table 1: Clinicopathological and molecular features of MB_{Grp4} compared to a non-MB_{Grp4} cohort. CLA = classic, DN/MBEN = desmoplastic nodular or medulloblastoma with extensive nodularity, LCA = large-cell/anaplastic, M0 = non-metastatic disease, M+ = metastatic disease, STR = subtotal resection, GTR = gross total resection, WCA = whole chromosome aberration-favourable risk, WCA-HR = whole chromosome aberration-high risk. Significance is shown by p values from either Fisher's exact tests, χ^2 test or t-test.

Supplementary Table 2: Table showing driver genes assessed for mutations in the study from both next-generation exome/panel sequencing and methylation focal CNV analysis, and their corresponding common critical biological pathways.

Supplementary Fig. 2: Time-dependent AUC at 5 years for a) number of WCAs and b) number of WCA gains c) number of WCA losses. d) Univariable Cox proportional hazards models of PFS for recurrent WCAs with incidences ≥15%. Kaplan-Meier PFS plots for prognostic WCAs, identified from univariable Cox regression e) gain of chromosome 7, f) loss of chromosome 8, g) loss of chromosome 11 and h) gain of chromosome 18. At-risk tables are shown in two-year increments with number of patients censored in parentheses with significance shown by *p* value generated from log-rank test. i) Time-dependent AUCs at 5 years for the top 5 optimal combinations of WCAs within MB_{Grp4}.

Supplementary Fig. 3: a) Unsupervised hierarchical clustering of all WCAs in MB_{Grp4}. WCAs with incidence ≥10% are annotated along with methylation subgroup. Numbers of whole chromosomal gains (red), losses (blue), and total changes (black) are also shown with increasing colour intensity indicating a higher number of changes. b) Association analyses of co-occurring WCAs (≥10% incidence). The strength of association is shown by odds ratio from Fisher's exact tests, with positive associations shown in blue and negative associations shown in red. Significance (*) is shown by p values from Fisher's exact tests.

Supplementary Fig. 4: a) tSNE visualisation of MB_{Grp4} second-generation methylation subgroup calls; non-classifiable (NC) samples still cluster with MB_{Grp4}. b) Violin plot showing age distribution of MB_{Grp4} second-generation methylation subgroups. Significance is shown by *p* value from ANOVA, subgroup 1 was not included in the analysis. Characterisation of MB_{Grp4} second-generation methylation subgroups showing c) all established clinicopathological characteristics, cytogenetic aberrations and d) genetic aberrations with a cohort-wide frequency of ≥5% or with a subgroup-specific frequency ≥10%. *MYC* amplifications are shown despite their low frequency. Significance is shown for both discovery and validation cohorts by *p* values from Fisher's exact or Kruskal-Wallis tests. Univariable Cox proportional hazards models of PFS in e) subgroup 5 and f) 8 for clinical and molecular features with ≥10% frequencies. M+ = metastatic disease, STR = subtotal resection, WCA = whole chromosome aberration, WCA-FR = whole chromosome aberration-favourable risk, HR = hazard ratio, CI = confidence interval.

Supplementary Fig. 5: Kaplan Meier plots for PFS in a) non-metastatic subgroup 6, b) subgroup 7 and c) non-metastatic subgroup 7 by WCA groups. At-risk tables are shown in two-year increments with number of patients censored in parentheses and significance shown by p value generated from log-rank tests. WCA-FR = whole chromosome aberration-favourable risk, WCA-HR = whole chromosome aberration-high risk.

Supplementary Fig. 6: Characterisation of WCA groups showing a) all established clinicopathological characteristics, cytogenetic aberrations and b) genetic aberrations with a cohort-wide frequency of ≥5% or with a subgroup-specific frequency ≥10%. *MYC* amplifications are shown despite their low frequency. Significance is shown for both discovery and validation cohorts by p values from Fisher's exact or Mann–Whitney U tests. c) Kaplan-Meier plot of PFS by MB_{Grp4} WCA status within the validation cohort, Cavalli *et al*. [3]. WCA = whole chromosome aberration, WCA-FR = whole chromosome aberration- favourable risk.

Supplementary Fig. 7: Calibration plots for a) the MB_{Grp4} risk-stratification model, b) Gajjar *et al*. [4] ($MB_{Grp3/4}$ scheme), c) Shih *et al*. [15] (cytogenetic scheme) and d) the current clinical scheme (SIOP-PNET-5-MB/ SIOP-HR-MB) [1, 8] at 5-year survival within the discovery cohort.

Supplementary Fig. 8: Calibration plots for a) Shih *et al*. [15] (cytogenetic scheme) and b) Gajjar *et al*. [4] ($MB_{Grp3/4}$ scheme) for survival at 5-years within the validation cohort (Northcott *et al*. [9]).

Supplementary Material References

1 Bailey S, André N, Gandola L, Massimino M, Rutkowski S, Clifford SC (2022) Clinical Trials in High-Risk Medulloblastoma: Evolution of the SIOP-Europe HR-MB Trial. Cancers (Basel) 14: Doi 10.3390/cancers14020374

2 Capper D, Jones DTW, Sill M, Hovestadt V, Schrimpf D, Sturm D, Koelsche C, Sahm F, Chavez L, Reuss DEet al (2018) DNA methylation-based classification of central nervous system tumours. Nature 555: 469-474 Doi 10.1038/nature26000

3 Cavalli FMG, Remke M, Rampasek L, Peacock J, Shih DJH, Luu B, Garzia L, Torchia J, Nor C, Morrissy ASet al (2017) Intertumoral Heterogeneity within Medulloblastoma Subgroups. Cancer Cell 31: 737-754.e736 Doi 10.1016/j.ccell.2017.05.005

4 Gajjar A, Robinson GW, Smith KS, Lin T, Merchant TE, Chintagumpala M, Mahajan A, Su J, Bouffet E, Bartels Uet al (2021) Outcomes by Clinical and Molecular Features in Children With Medulloblastoma Treated With Risk-Adapted Therapy: Results of an International Phase III Trial (SJMB03). Journal of Clinical Oncology 39: 822-835 Doi 10.1200/jco.20.01372

5 Goschzik T, Schwalbe EC, Hicks D, Smith A, Zur Muehlen A, Figarella-Branger D, Doz F, Rutkowski S, Lannering B, Pietsch Tet al (2018) Prognostic effect of whole chromosomal aberration signatures in standard-risk, non-WNT/non-SHH medulloblastoma: a retrospective, molecular analysis of the HIT-SIOP PNET 4 trial. Lancet Oncol 19: 1602-1616 Doi 10.1016/S1470-2045(18)30532-1

6 Ho B, Johann PD, Grabovska Y, De Dieu Andrianteranagna MJ, Yao F, Frühwald M, Hasselblatt M, Bourdeaut F, Williamson D, Huang Aet al (2019) Molecular subgrouping of atypical teratoid/rhabdoid tumors—a reinvestigation and current consensus. Neuro-Oncology 22: 613-624 Doi 10.1093/neuonc/noz235

7 Lindsey JC, Schwalbe EC, Potluri S, Bailey S, Williamson D, Clifford SC (2014) TERT promoter mutation and aberrant hypermethylation are associated with elevated expression in medulloblastoma and characterise the majority of non-infant SHH subgroup tumours. Acta Neuropathol 127: 307-309 Doi 10.1007/s00401-013-1225-3

8 Mynarek M, Milde T, Padovani L, Janssens GO, Kwiecien R, Mosseri V, Clifford SC, Doz F, Rutkowski S (2021) SIOP PNET5 MB Trial: History and Concept of a Molecularly Stratified Clinical Trial of Risk-Adapted Therapies for Standard-Risk Medulloblastoma. Cancers (Basel) 13: Doi 10.3390/cancers13236077

9 Northcott PA, Buchhalter I, Morrissy AS, Hovestadt V, Weischenfeldt J, Ehrenberger T, Gröbner S, Segura-Wang M, Zichner T, Rudneva VAet al (2017) The whole-genome landscape of medulloblastoma subtypes. Nature 547: 311-317 Doi 10.1038/nature22973

10 Northcott PA, Lee C, Zichner T, Stütz AM, Erkek S, Kawauchi D, Shih DJ, Hovestadt V, Zapatka M, Sturm Det al (2014) Enhancer hijacking activates GFI1 family oncogenes in medulloblastoma. Nature 511: 428-434 Doi 10.1038/nature13379

11 Ramaswamy V, Remke M, Bouffet E, Bailey S, Clifford SC, Doz F, Kool M, Dufour C, Vassal G, Milde Tet al (2016) Risk stratification of childhood medulloblastoma in the molecular era: the current consensus. Acta Neuropathol 131: 821-831 Doi 10.1007/s00401-016-1569-6

12 Richardson S, Hill RM, Kui C, Lindsey JC, Grabovksa Y, Keeling C, Pease L, Bashton M, Crosier S, Vinci Met al (2021) Emergence and maintenance of actionable genetic drivers at medulloblastoma relapse. Neuro-Oncology 24: 153-165 Doi 10.1093/neuonc/noab178

13 Schwalbe EC, Hicks D, Rafiee G, Bashton M, Gohlke H, Enshaei A, Potluri S, Matthiesen J, Mather M, Taleongpong Pet al (2017) Minimal methylation classifier (MIMIC): A novel method for derivation and rapid diagnostic detection of disease-associated DNA methylation signatures. Scientific Reports 7: Doi 10.1038/s41598-017-13644-1

14 Schwalbe EC, Lindsey JC, Nakjang S, Crosier S, Smith AJ, Hicks D, Rafiee G, Hill RM, Iliasova A, Stone Tet al (2017) Novel molecular subgroups for clinical classification and outcome

prediction in childhood medulloblastoma: a cohort study. Lancet Oncol 18: 958-971 Doi 10.1016/s1470-2045(17)30243-7
15 Shih DJ, Northcott PA, Remke M, Korshunov A, Ramaswamy V, Kool M, Luu B, Yao Y, Wang X, Dubuc AMet al (2014) Cytogenetic prognostication within medulloblastoma subgroups. J Clin Oncol 32: 886-896 Doi 10.1200/JCO.2013.50.9539
